# Supplementary material for: Leveraging data-driven self-consistency for high-fidelity gene expression recovery
Source: Nat Commun. 2022 Nov 21;13:7142. doi: 10.1038/s41467-022-34595-w (PMC9681852; doi:10.1038/s41467-022-34595-w)
Supplement: Supplementary file 1 — Supplementary Information [file 41467_2022_34595_MOESM1_ESM.pdf]

Contents

1 Modeling of gene expression data 3

2 IDH-mutant gliomas 12

3 Single-cell analysis in pediatric midline gliomas 14

4 Melanoma intra-tumor heterogeneity 16

5 Div-Seq data analysis 18

6 Clustering of intestinal immune cell atlas 20

7 Computational speed 23

8 Distribution of denoised data learned when different number of data points are used 28

9 HCL and MCA datasets 30

10 Slingshot and TSCAN analysis of imputed data from different methods 35

11 Batch effect correction by SERM 37

12 Statistics of reference and observed datasets 41

13 Analysis of Tabula Muris (TM) dataset 43

1. Modeling of gene expression data

To illustrate the appropriateness of the used distributions and global consistency for modeling the gene expression values, we divide 100 gene expression data matrices simulated by Splatter simulator into four regions. We then fit the learned distribution with λ parameter to the histograms of the data of 4 regions separately and of the whole matrix. We found that the λ parameters for each dataset were very similar with less than 9% standard deviation from the mean value. As an example, we show the fitted curve for a twenty class dropout-free gene expression data matrix simulated with Splatter simulator. The obtained λ parameter for five data fits was 9.34, 8.61, 8.61, 8.97 and 8.63, respectively. The fitted histograms are shown in Fig. S1. The good quality of fitted exponential distribution to the histograms proves the appropriateness of the learned distribution. Moreover, the closeness of the obtained distribution parameters for the five different ROIs supports the notion of global consistency of data in gene expression matrices.

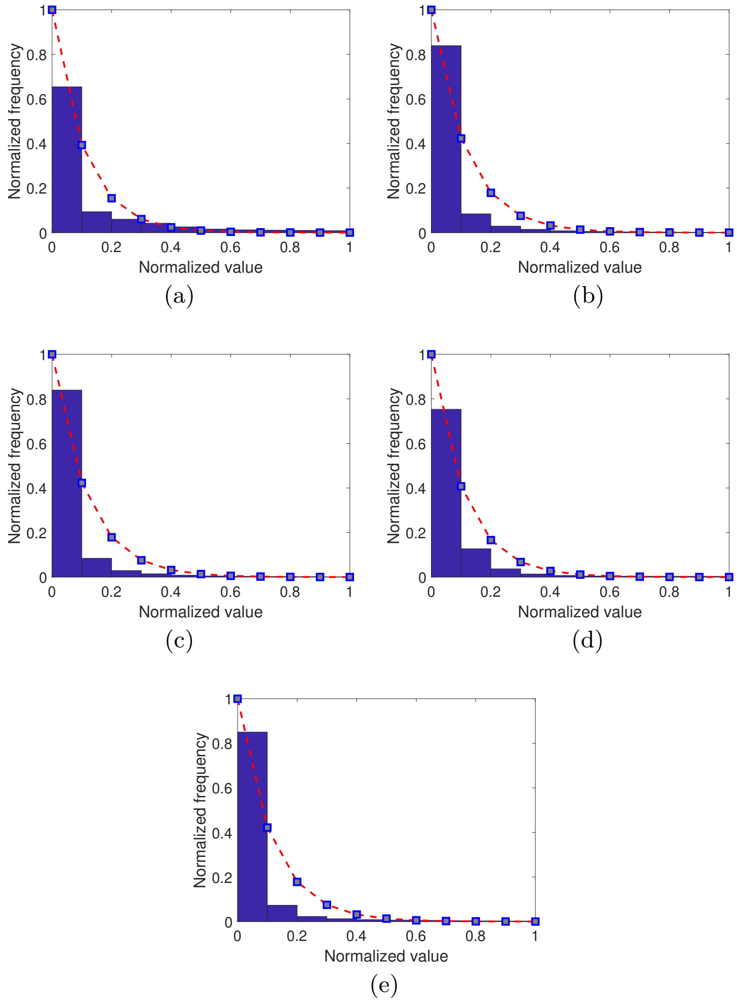

Fig. S1. (a)-(d) Genomic data with 20 classes simulated with Splatter simulator without dropout. Classes 1-20 have the same rate parameter of 0.9 and shape parameters of 0.10, 0.11, 0.15, 0.16, 0.20, 0.21, 0.25, 0.26, 0.30, 0.31, 0.35, 0.36, 0.40, 0.41, 0.50, 0.52, 0.70, 0.71, 0.80 and 0.805, respectively. Other simulation parameters are set at default numbers. For SERM analysis, the whole gene expression matrix (of size 20,000 $\times$ 300) is divided into 4 regions. The distribution of data in the 4 regions (region 1-row 1 to row 10,000, column 1 to 150; region 2-row 10001 to row 20,000, column 1 to 150; region 3-row 1 to row 10,000, column 151 to 300; region 4-row 10,001 to row 20,000, column 151 to 300) is shown in (a)-(d), respectively with learned exponential fit. (e) Global distribution of gene expression in expression matrix with learned exponential fit. The number of histogram bin was set to 100. The histogram has non-zero values in first few bins. The exponential curve was fit to values corresponding to the first ten bins as shown in the figure. The x-axis and y-axis are normalized to 1. The λ parameter for the exponential distribution for these five data fits were 9.34, 8.61, 8.61, 8.97 and 8.63, respectively.

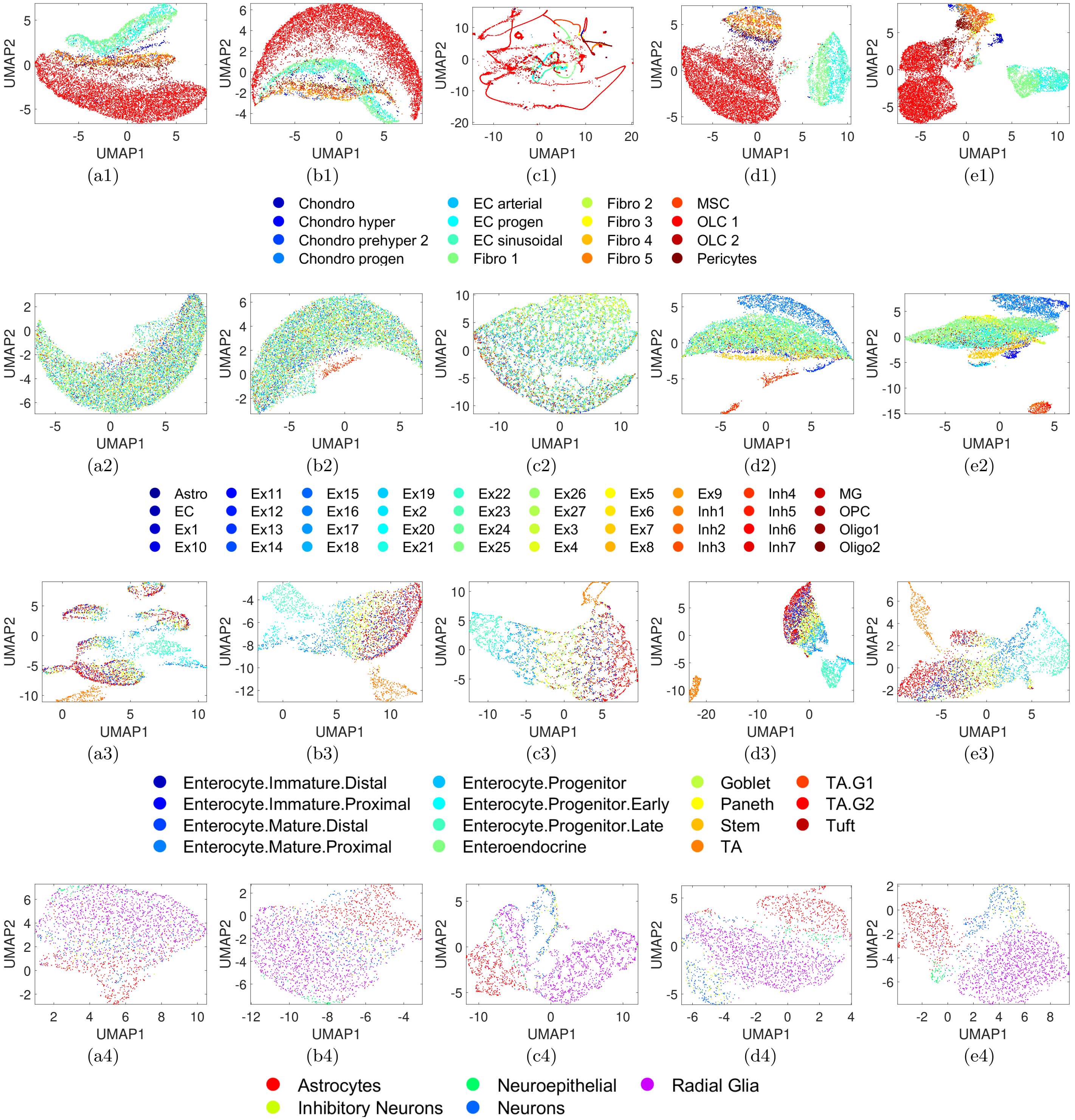

Fig. S2. UMAP visualizations of imputed data from AutoImpute (a), DeepImpute (b), SAUCIE (c), DCA (d), and scVI (e) for (1) cellular taxonomy, (2) mammalian brain, (3) mouse intestinal epithelium and (4) 3D neural tissue datasets.

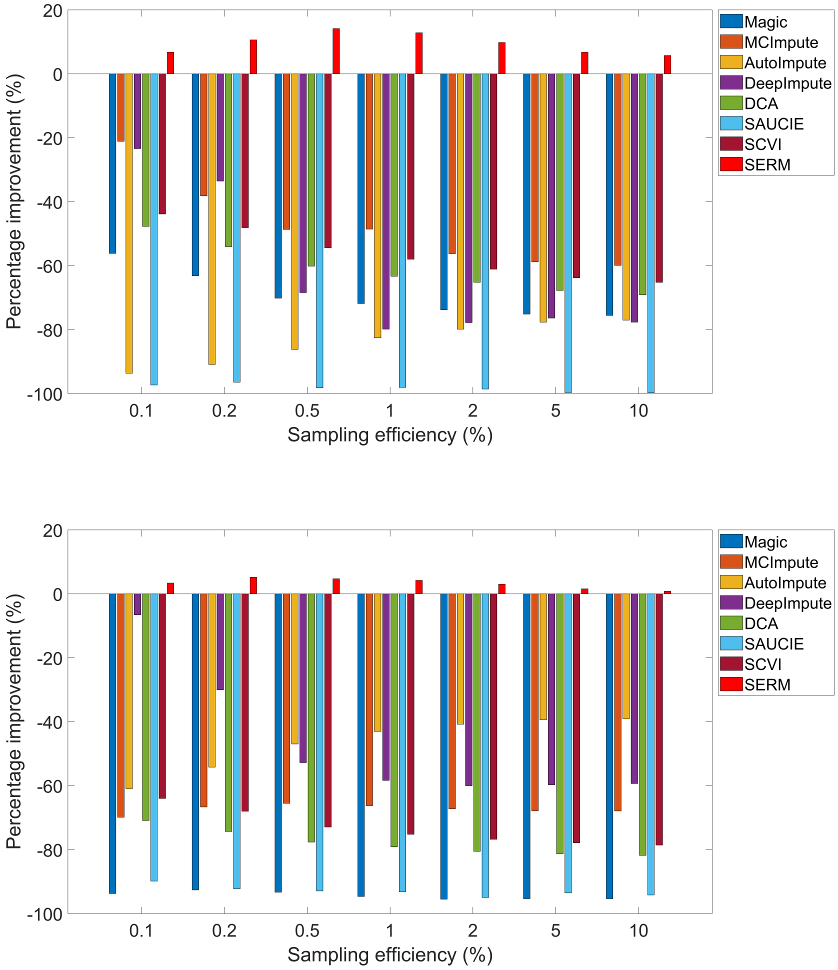

Fig. S3. Percent improvement in mean Pearson coefficient of the imputed data (in comparison to the observed data) by eight different techniques for cellular taxonomy (row 1) and mammalian brain (row 2) datasets. The sampling efficiencies (0.1%-10%) to create the observed data are shown in x-axis. See 'Computation of Pearson coefficient' subsection of the Methods section for the calculation process of the percent improvement.

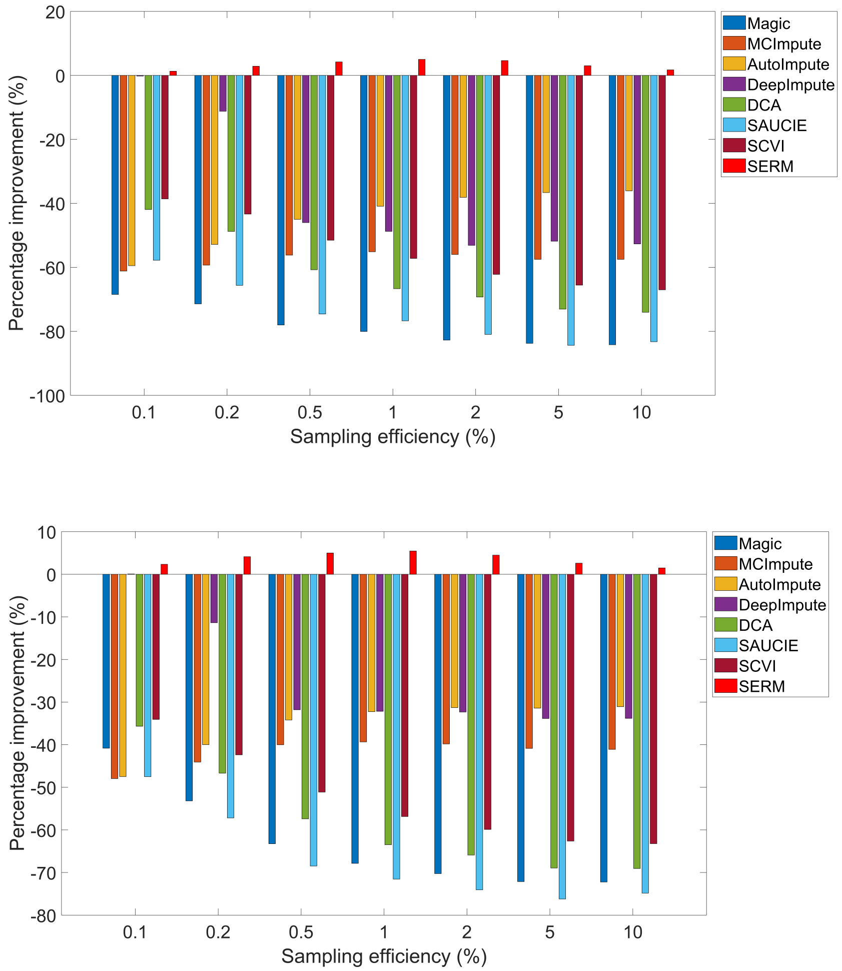

Fig. S4. Percent improvement in mean Pearson coefficient of the imputed data (in comparison to the observed data) by eight different techniques for mouse intestinal epithelium (row 1) and 3D neural tissue (row 2) datasets. The sampling efficiencies (0.1%-10%) to create the observed data are shown in x-axis.

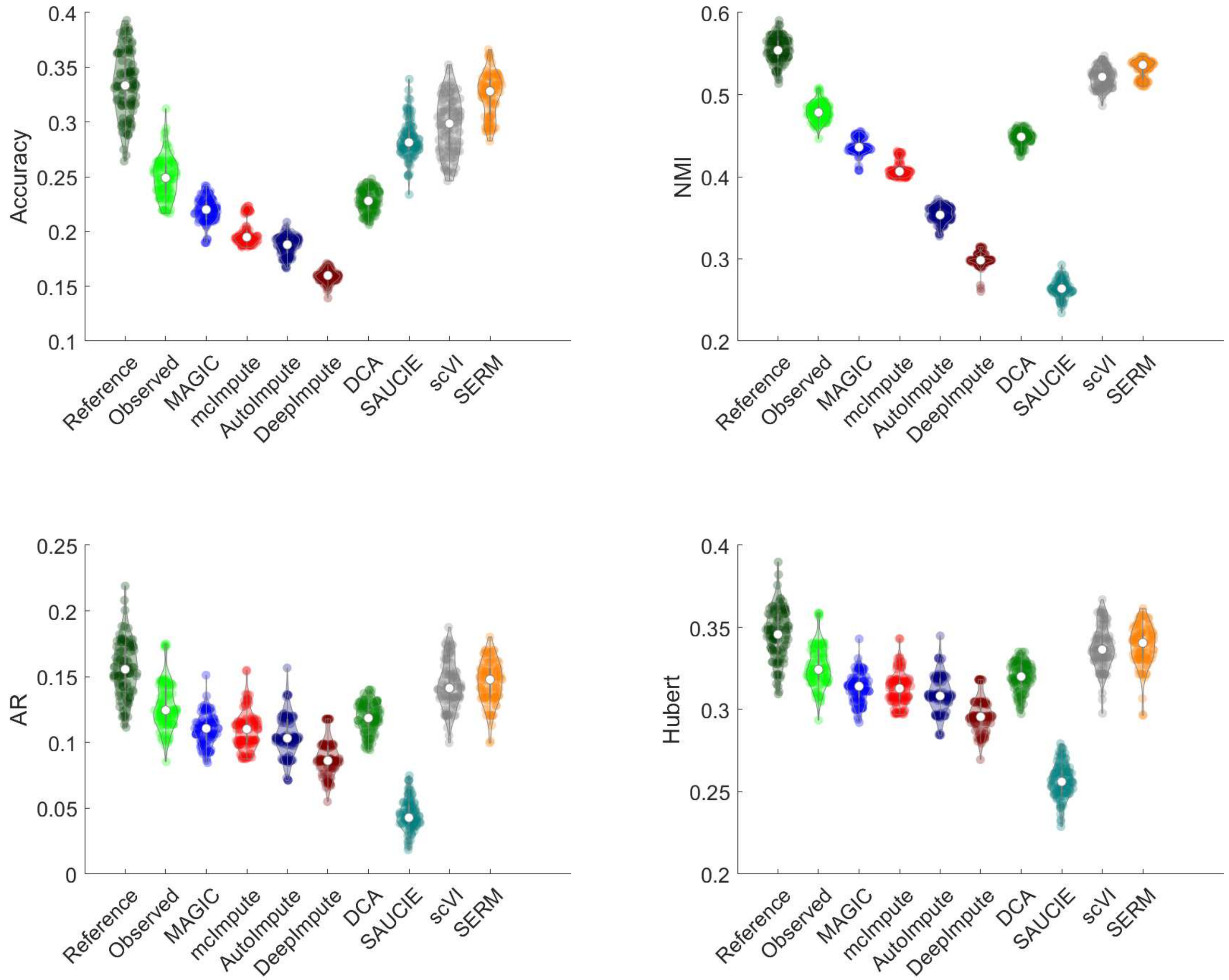

Fig. S5. Cluster quality indices (accuracy, NMI, AR, and Hubert) of UMAP visualizations of data imputed by different techniques for cellular taxonomy dataset. The spread of the violin represent the deviation of the indices for 100 different initializations of k-means clustering.

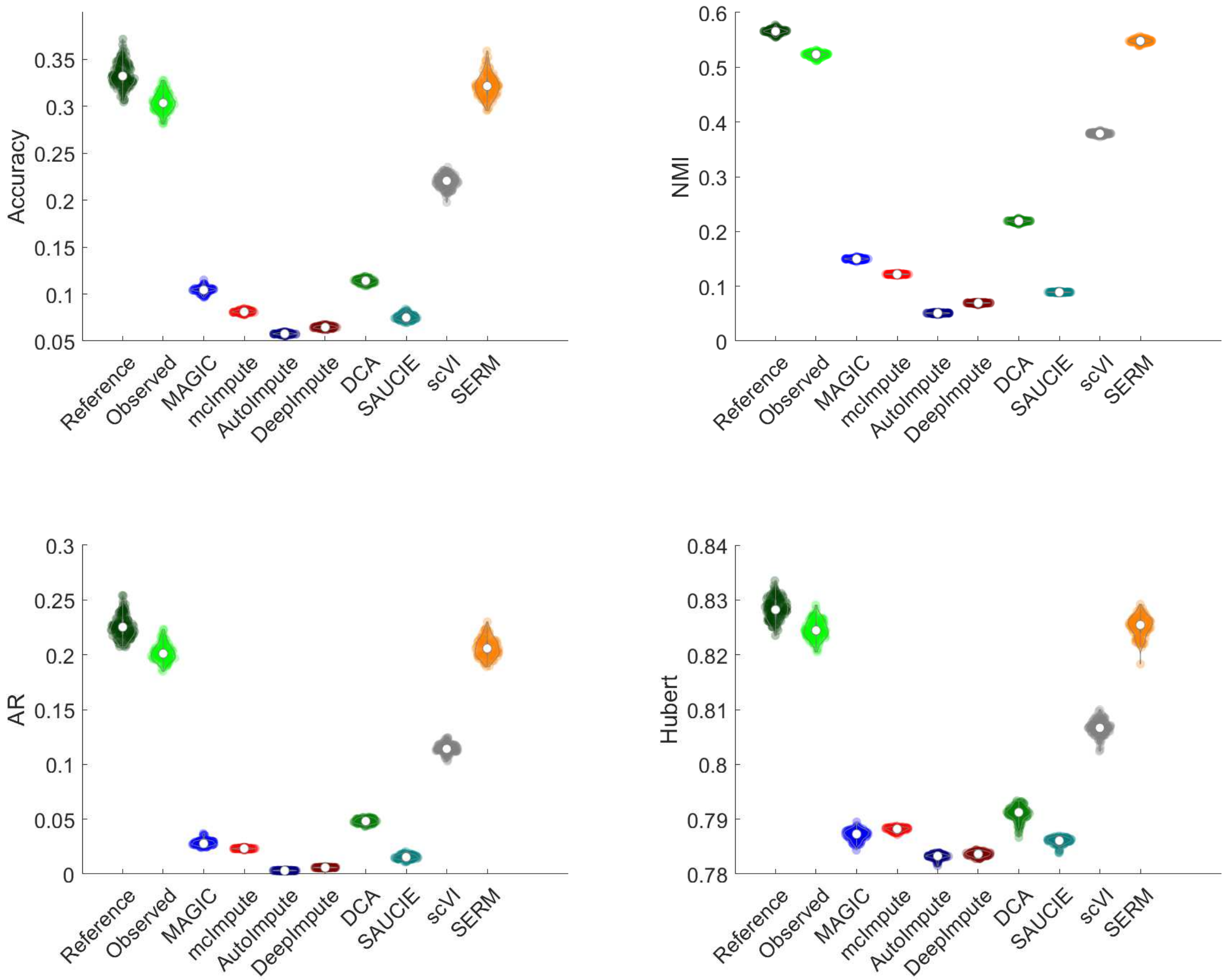

Fig. S6. Cluster quality indices (accuracy, NMI, AR, and Hubert) of UMAP visualizations of data imputed by different techniques for mammalian brain dataset. The spread of the violin represent the deviation of the indices for 100 different initializations of k-means clustering.

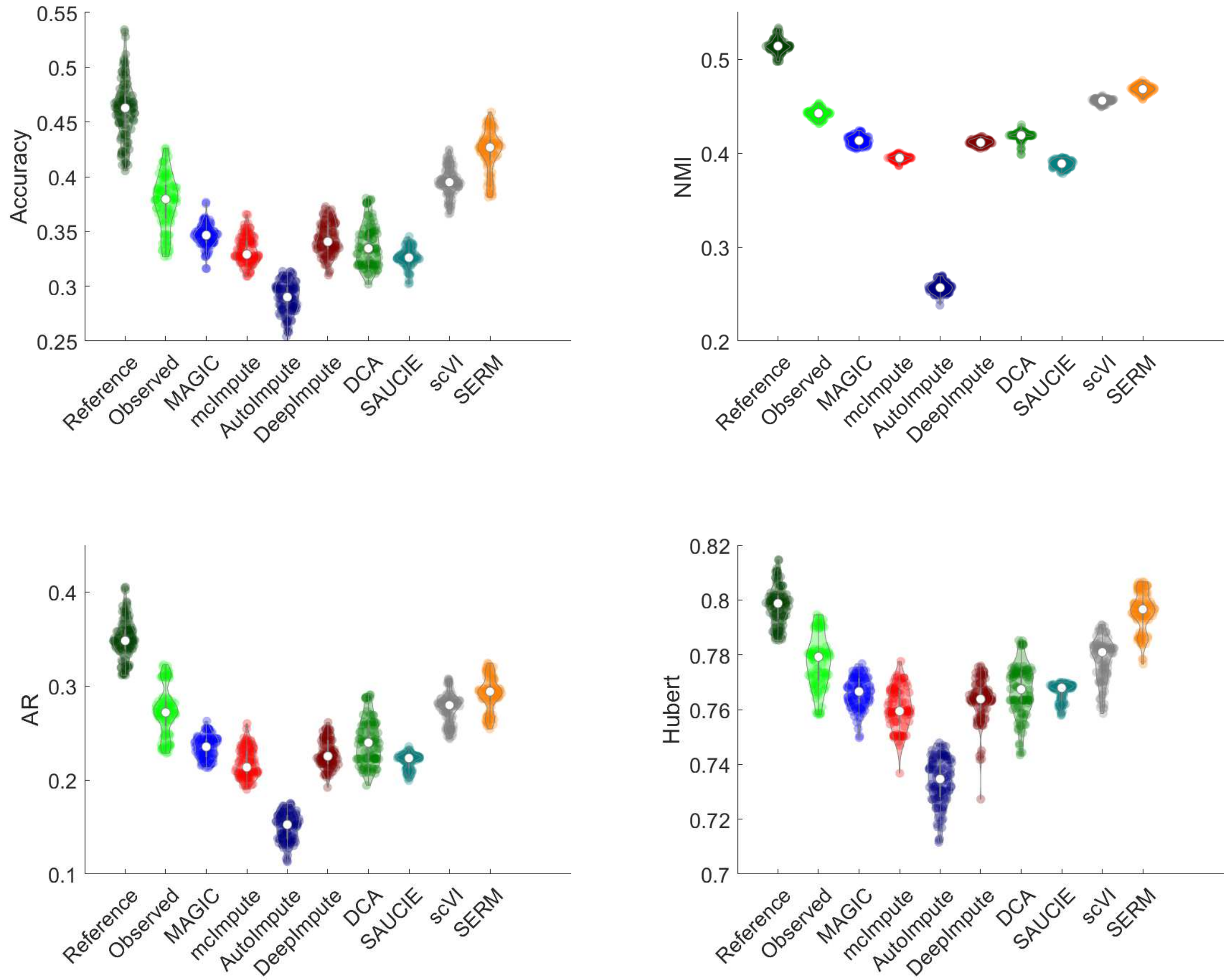

Fig. S7. Cluster quality indices (accuracy, NMI, AR, and Hubert) of UMAP visualizations of data imputed by different techniques for mouse intestinal epithelium dataset. The spread of the violin represent the deviation of the indices for 100 different initializations of k-means clustering.

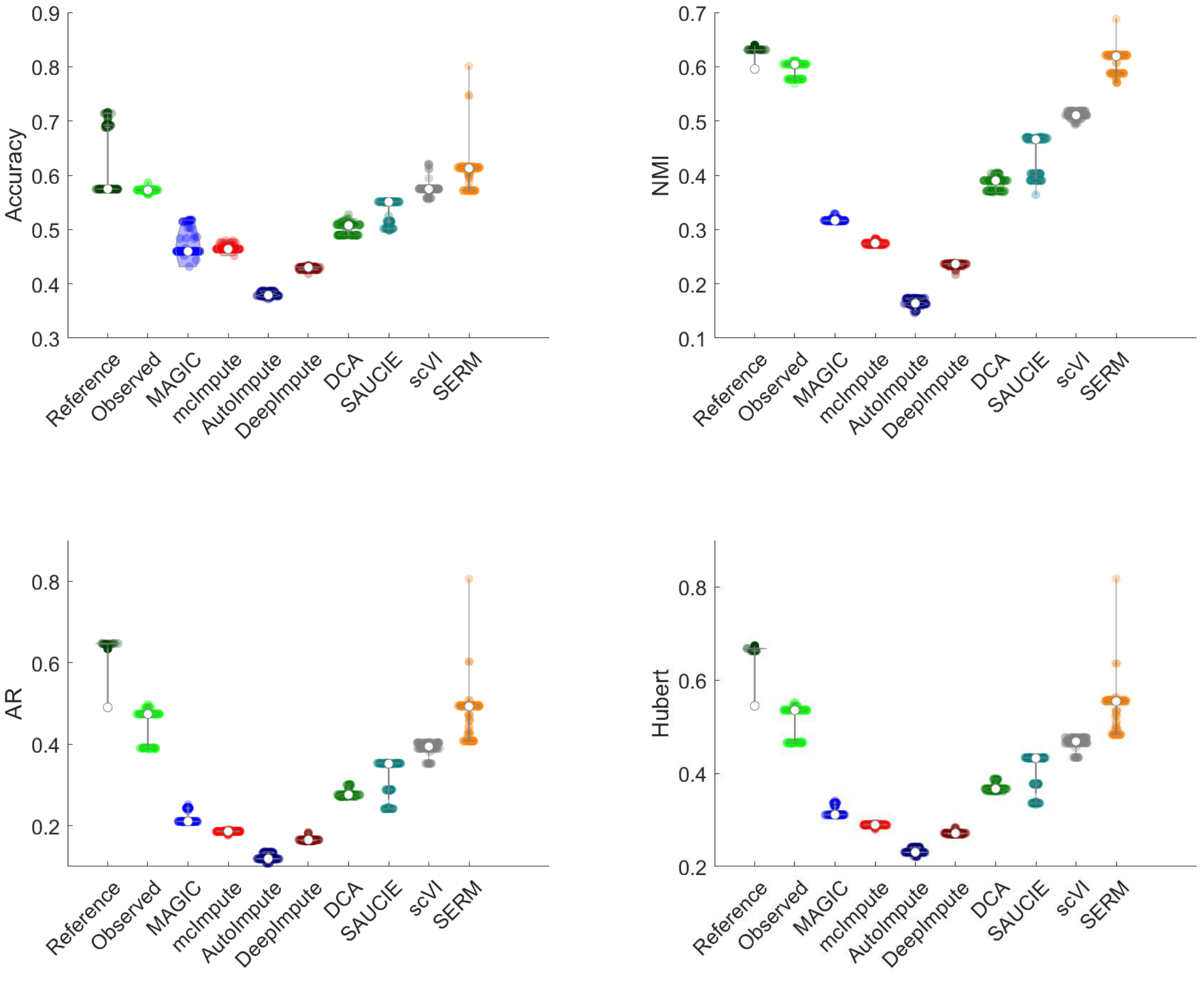

Fig. S8. Cluster quality indices (accuracy, NMI, AR, and Hubert) of UMAP visualizations of data imputed by different techniques for 3D neural tissue dataset. The spread of the violin represent the deviation of the indices for 100 different initializations of k-means clustering.

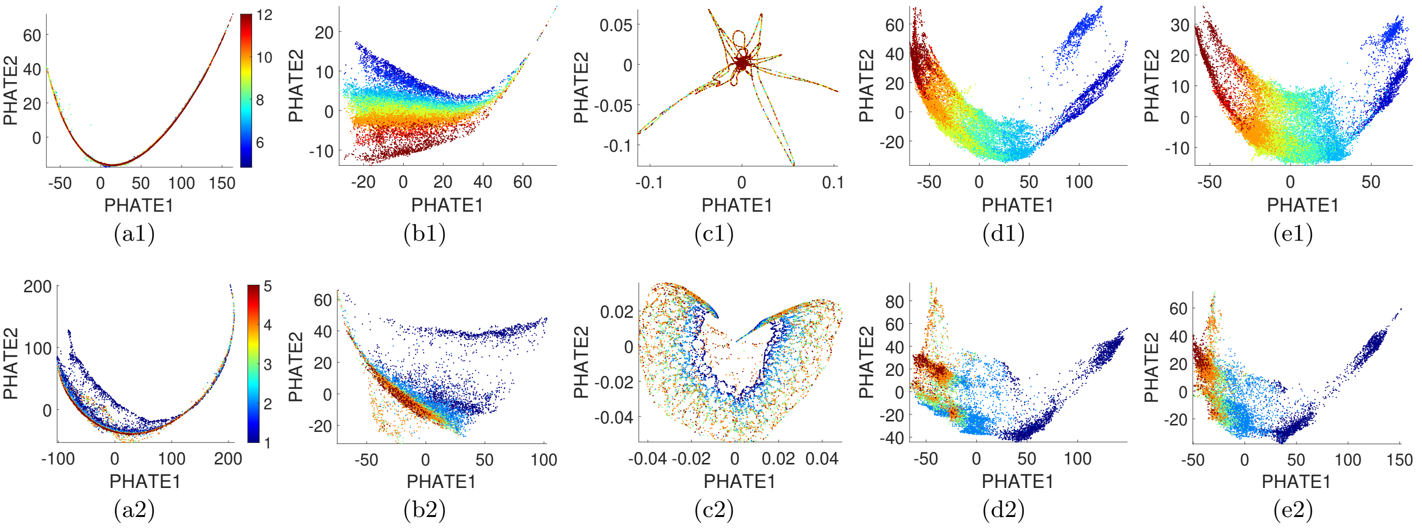

Fig. S9. PHATE visualizations of imputed data from AutoImpute (a), DeepImpute (b), SAUCIE (c), DCA (d), and scVI (e) for (1) zebrafish development and (2) EB differentiation datasets.

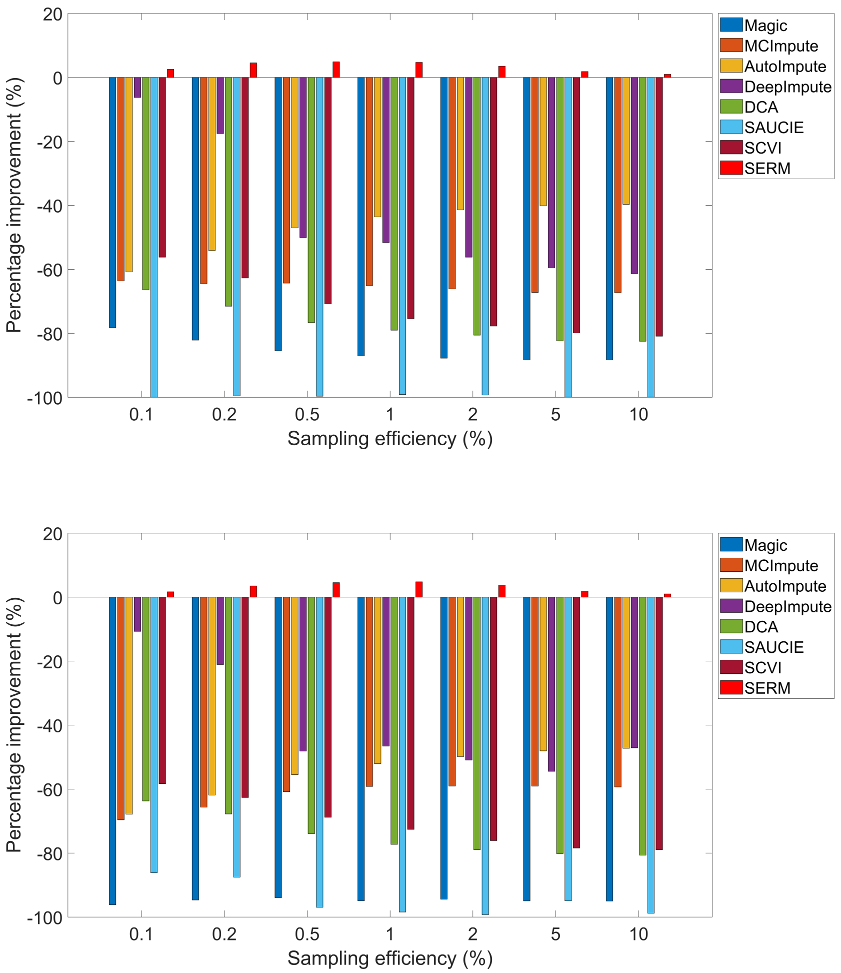

Fig. S10. Percent improvement in mean Pearson coefficient of the imputed data (in comparison to the observed data) by eight different techniques for zebrafish development (row 1) and EB differentiation (row 2) datasets. The sampling efficiencies (0.1%-10%) to create the observed data are shown in x-axis. See 'Computation of Pearson coefficient' subsection of the Methods section for the calculation process of the percent improvement.

2. IDH-mutant gliomas

Tumor subclasses differ according to the genotypes of malignant cells as well as the tumor microenvironment (TME)(1). The authors of Ref. (1) dissected these influences in isocitrate dehydrogenase (IDH)–mutant gliomas by combining 14,226 scRNA-seq profiles from 16 patient samples with bulk RNA-seq profiles from 165 patient samples. Differences in bulk profiles between IDH-mutant astrocytoma and oligodendroglioma can be primarily explained by distinct TME and signature genetic events, whereas both tumor types share similar developmental hierarchies and lineages of glial differentiation.

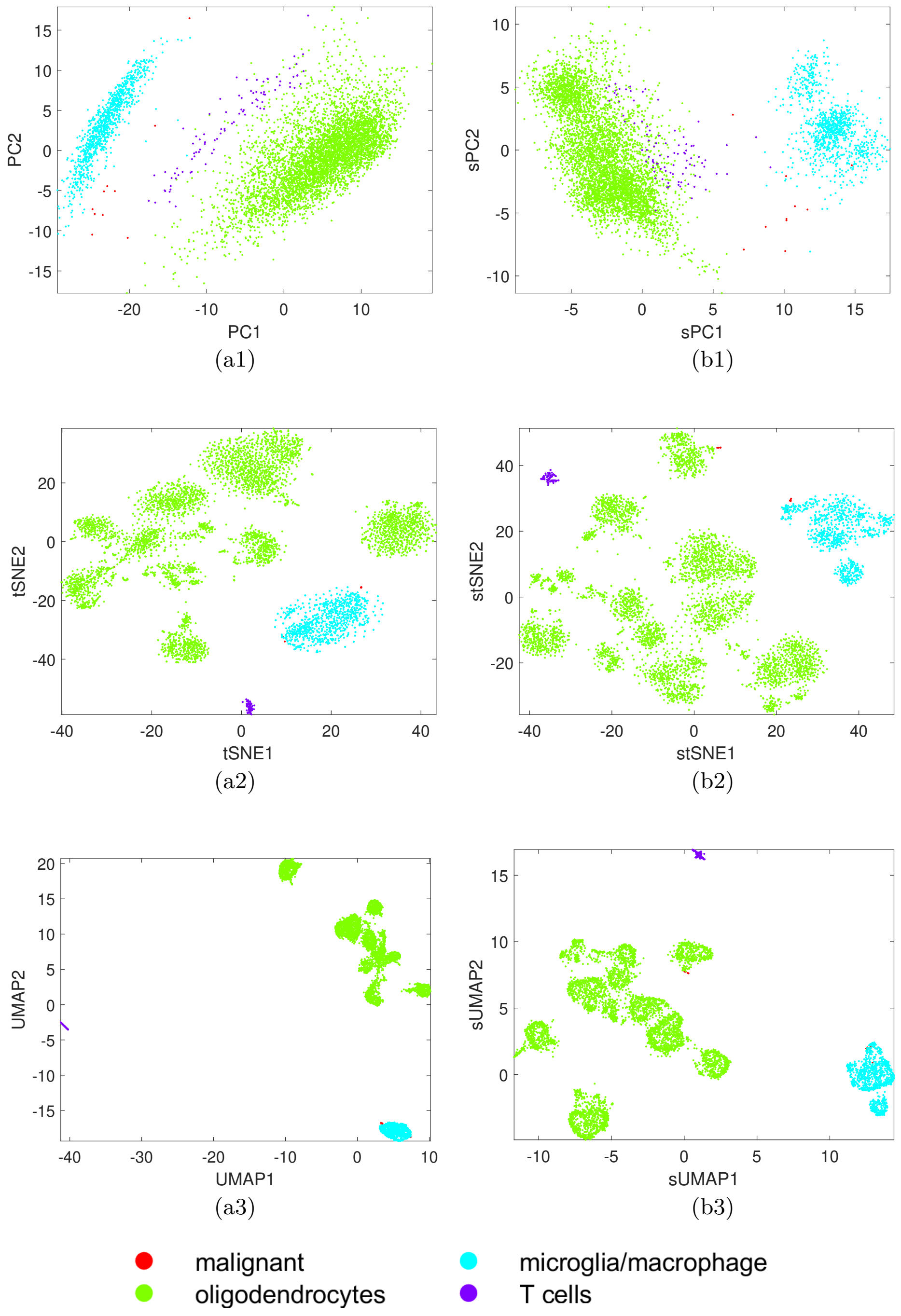

Fig. S11. IDH-mutant gliomas dataset. (a1,a2,a3) PCA, t-SNE and UMAP results without any imputation. (b1,b2,b3) PCA, t-SNE and UMAP results after SERM imputation.

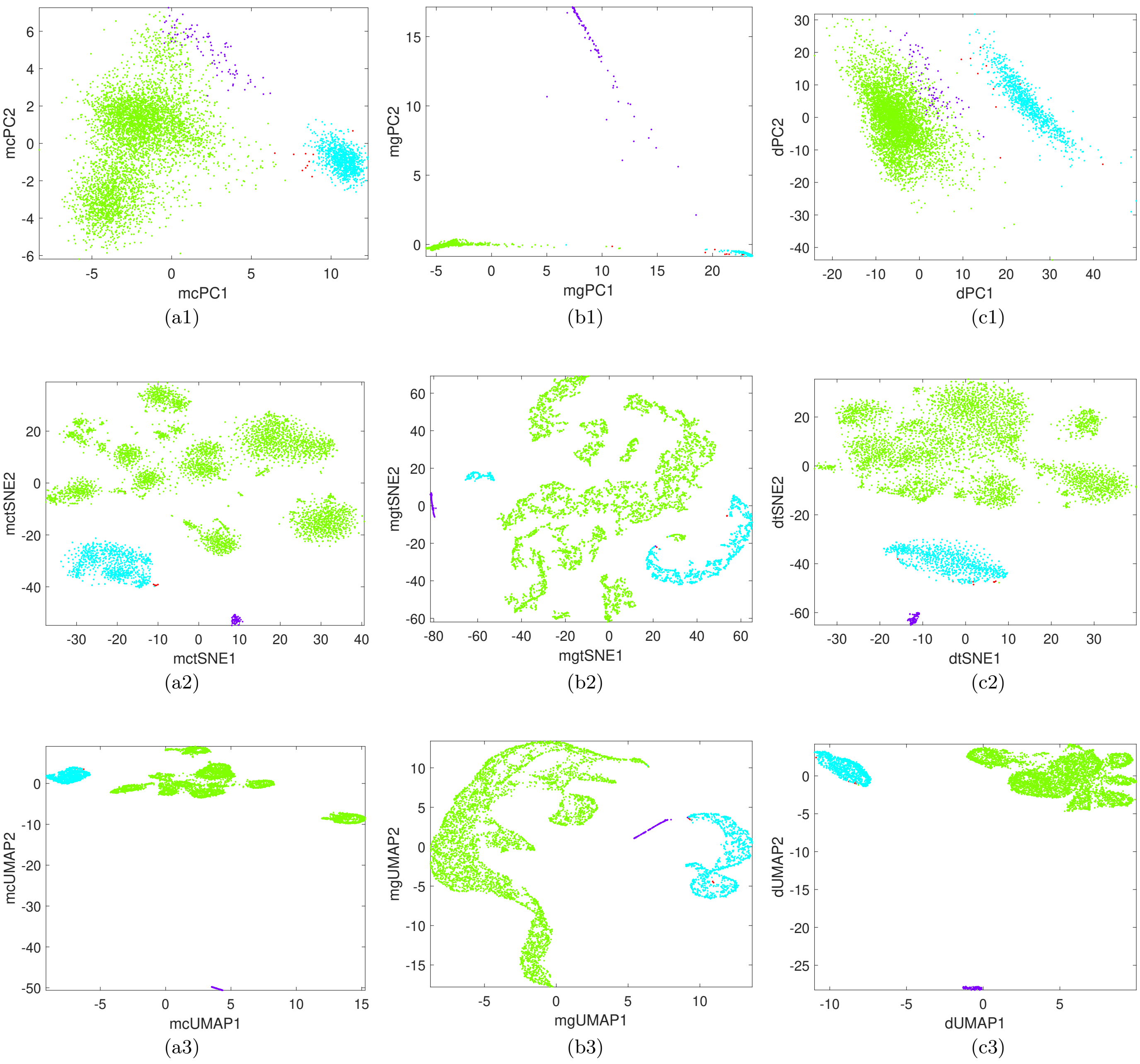

Fig. S12. IDH-mutant gliomas dataset. (a1,a2,a3) PCA, t-SNE and UMAP results with mcImpute imputation. (b1,b2,b3) PCA, t-SNE and UMAP results after MAGIC imputation. (c1,c2,c3) PCA, t-SNE and UMAP results after DeepImpute imputation. Color legends are shown in Fig. S11.

3. Single-cell analysis in pediatric midline gliomas

Gliomas with histone H3 lysine27-to-methionine mutations (H3K27M-glioma) arise primarily in the midline of the central nervous system of young children, suggesting a cooperation between genetics and cellular context in tumorigenesis (2). While the genetics of H3K27M-glioma are well-characterized, their cellular architecture remains uncharted. In this dataset, the authors performed scRNA-seq in 3,321 cells from six primary H3K27M-glioma and matched models. This study characterizes oncogenic and developmental programs in H3K27M-glioma at single-cell resolution and across genetic subclones, suggesting potential therapeutic targets in this disease.

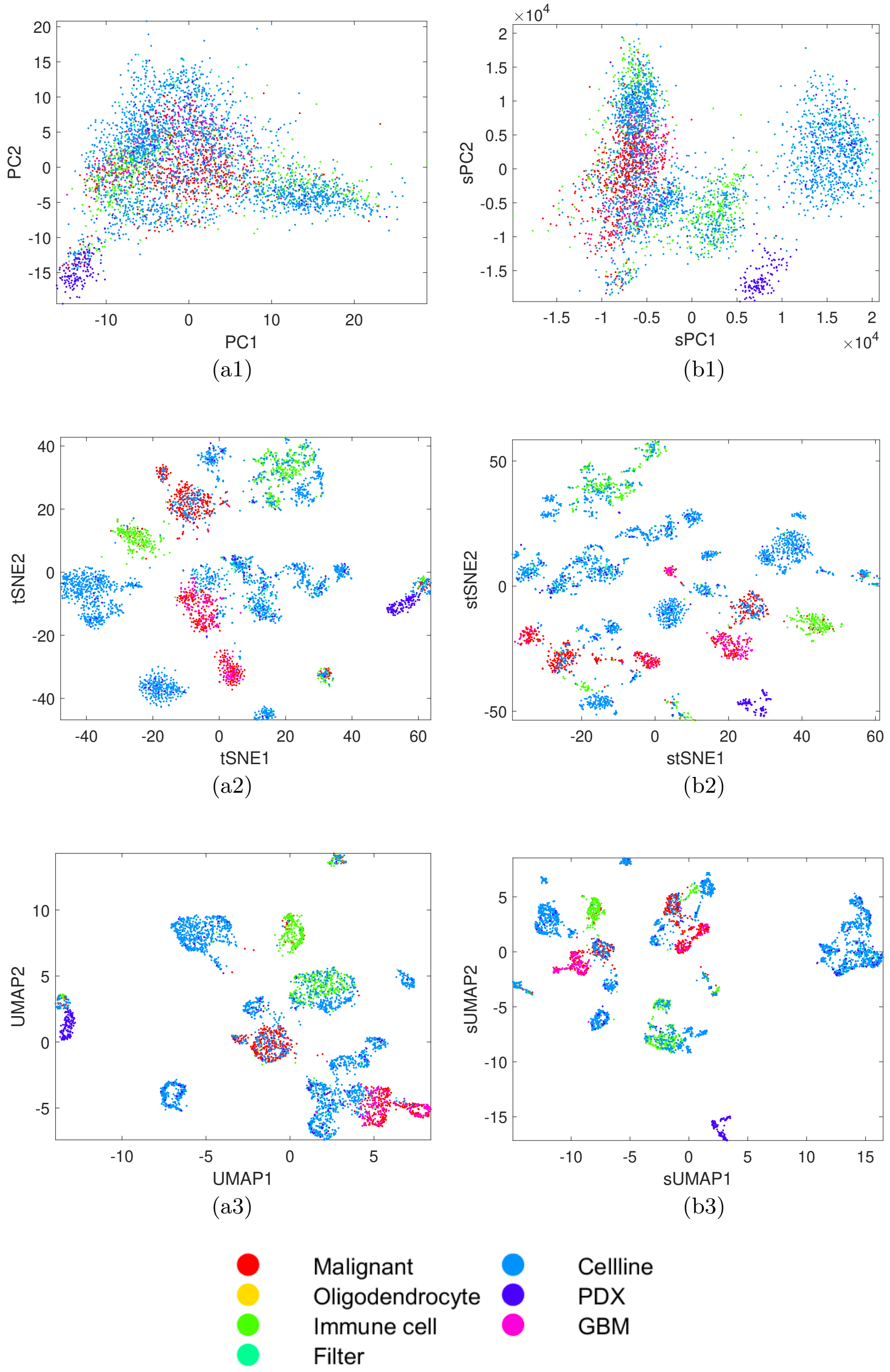

Fig. S13. Pediatric midline gliomas dataset. (a1,a2,a3) PCA, t-SNE and UMAP results without any imputation. (b1,b2,b3) PCA, t-SNE and UMAP results after SERM imputation.

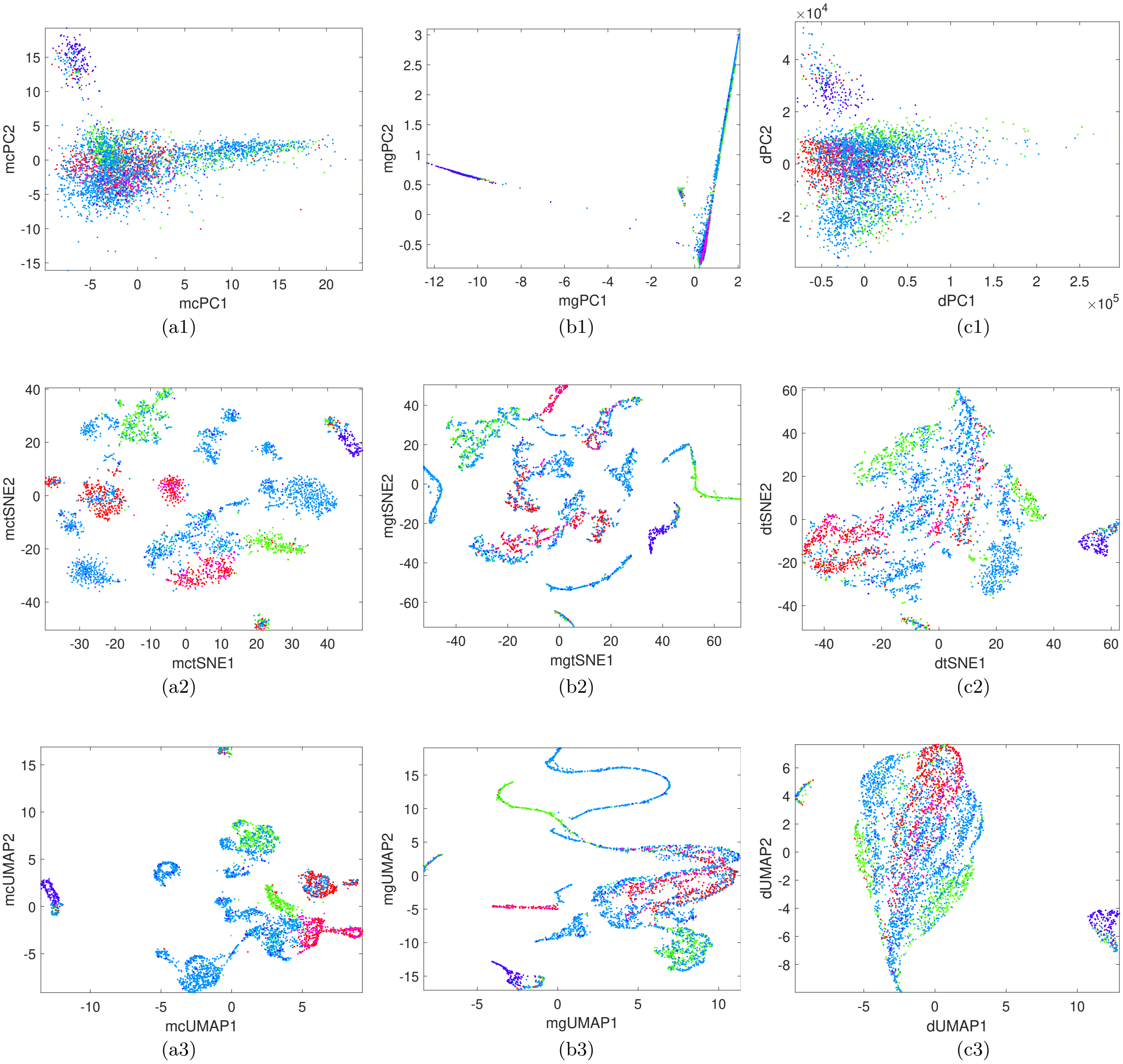

Fig. S14. Pediatric midline gliomas dataset. (a1,a2,a3) PCA, t-SNE and UMAP results with mcImpute imputation. (b1,b2,b3) PCA, t-SNE and UMAP results after MAGIC imputation. (c1,c2,c3) PCA, t-SNE and UMAP results after DeepImpute imputation. Color legends are shown in Fig. S13.

4. Melanoma intra-tumor heterogeneity

To explore the distinct genotypic and phenotypic states of melanoma tumors, the authors of Ref. (3) applied scRNA-seq to 4645 single cells isolated from 19 patients, profiling malignant, immune, stromal, and endothelial cells. Single-cell analyses suggested distinct tumor microenvironmental patterns, including cell-to-cell interactions. Analysis of tumor-infiltrating T cells revealed exhaustion programs, their connection to T cell activation and clonal expansion, and their variability across patients. Overall, in this dataset, the cellular ecosystem of tumors began to unravel and how single-cell genomics offers insights with implications for both targeted and immune therapies.

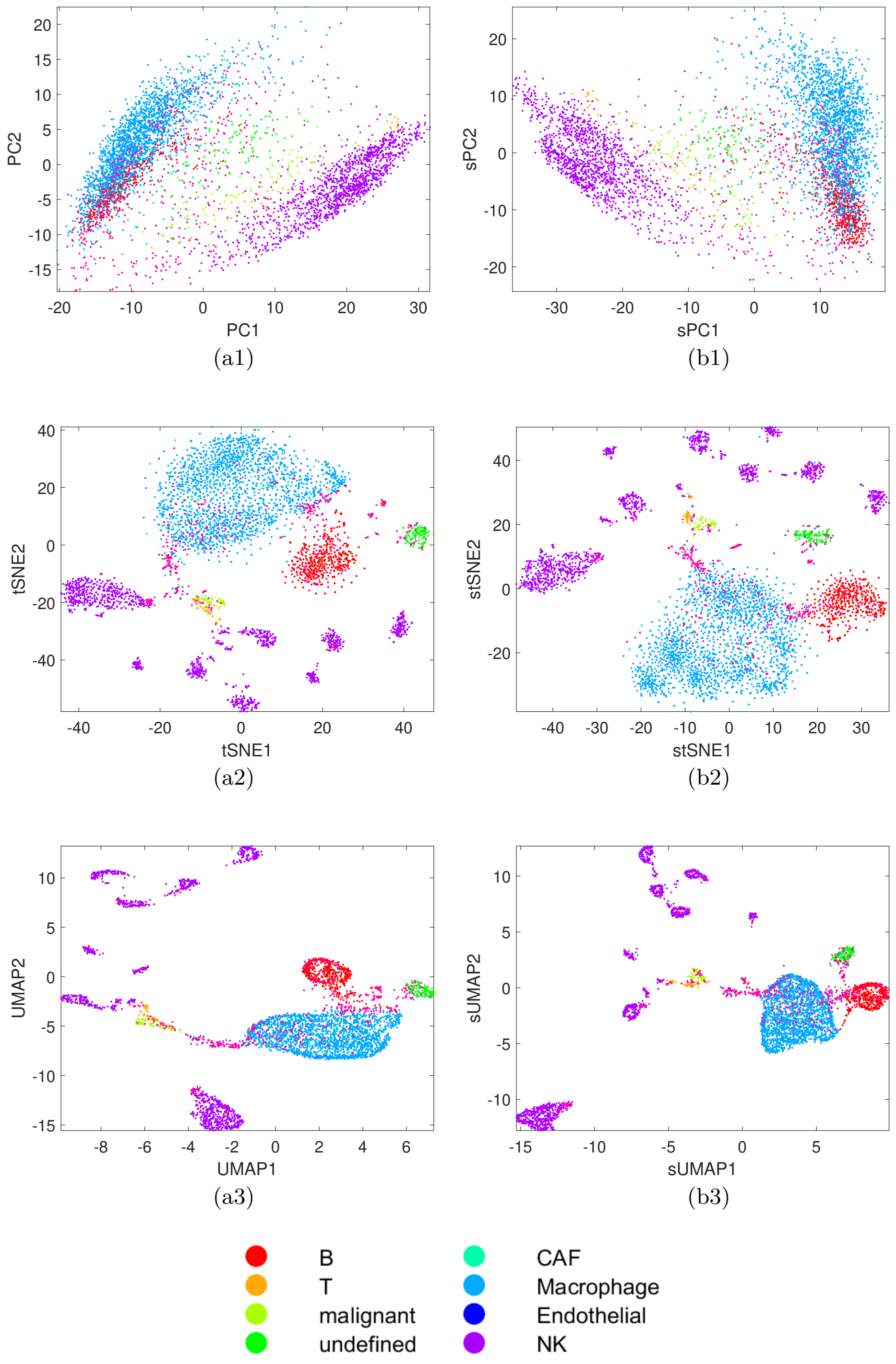

Fig. S15. Melanoma dataset. (a1,a2,a3) PCA, t-SNE and UMAP results without any imputation. (b1,b2,b3) PCA, t-SNE and UMAP results after SERM imputation.

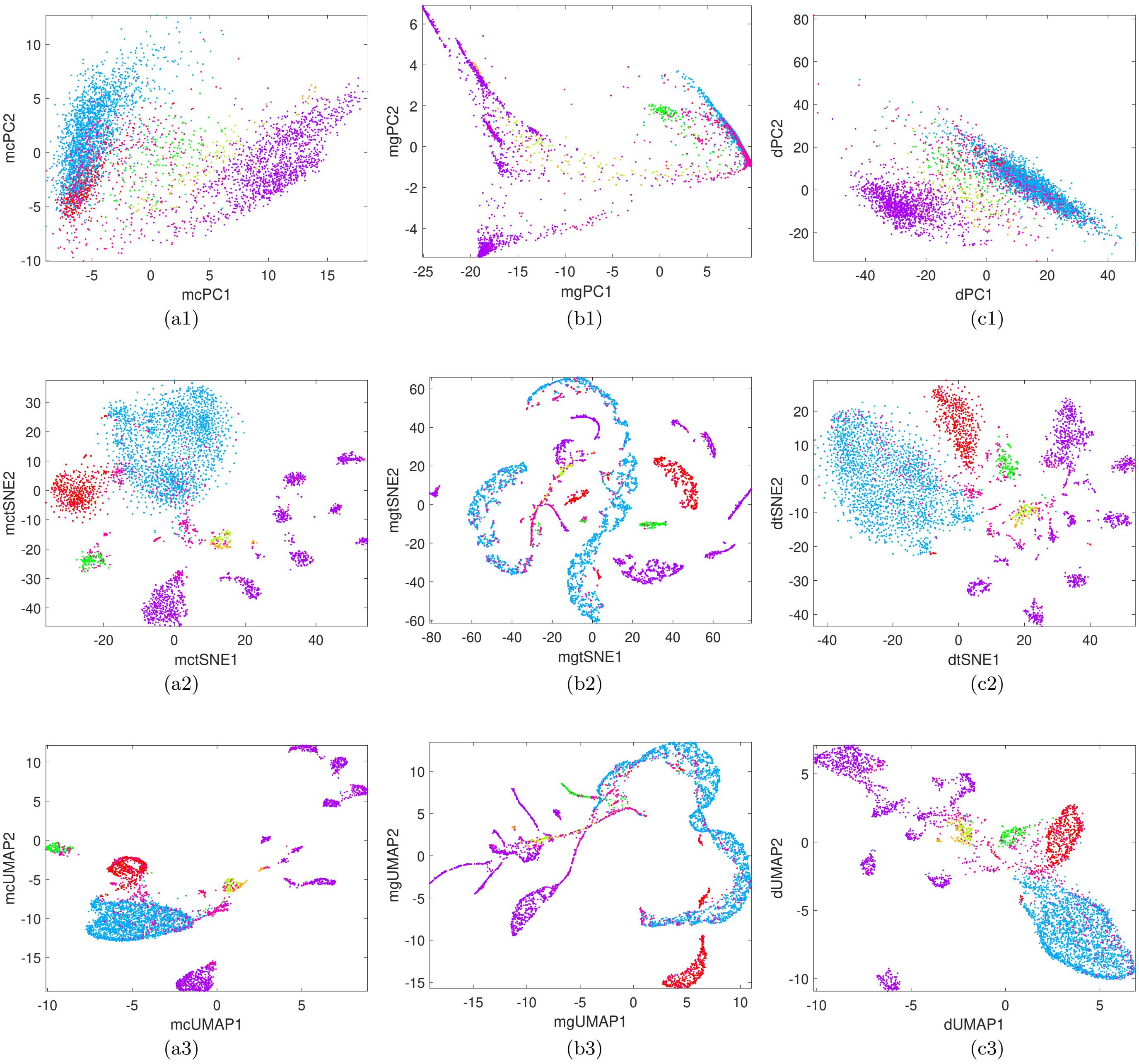

Fig. S16. Melanoma dataset. (a1,a2,a3) PCA, t-SNE and UMAP results with mcImpute imputation. (b1,b2,b3) PCA, t-SNE and UMAP results after MAGIC imputation. (c1,c2,c3) PCA, t-SNE and UMAP results after DeepImpute imputation. Color legends are shown in Fig. S15.

5. Div-Seq data analysis

ScRNA-seq provides rich information about cell types and states (4). However, it is difficult to capture rare dynamic processes, such as adult neurogenesis, because isolation of rare neurons from adult tissue is challenging and markers for each phase are limited. Div-Seq technique is developed to solve this problem, which combines scalable single-nucleus RNA-Seq (sNuc-Seq) with pulse labeling of proliferating cells. Using sNuc-Seq, the authors of Ref. (4) analyzed 1,367 single nuclei from hippocampal anatomical sub-regions from adult mice, including enrichment of genetically-tagged lowly abundant GABAergic neurons.

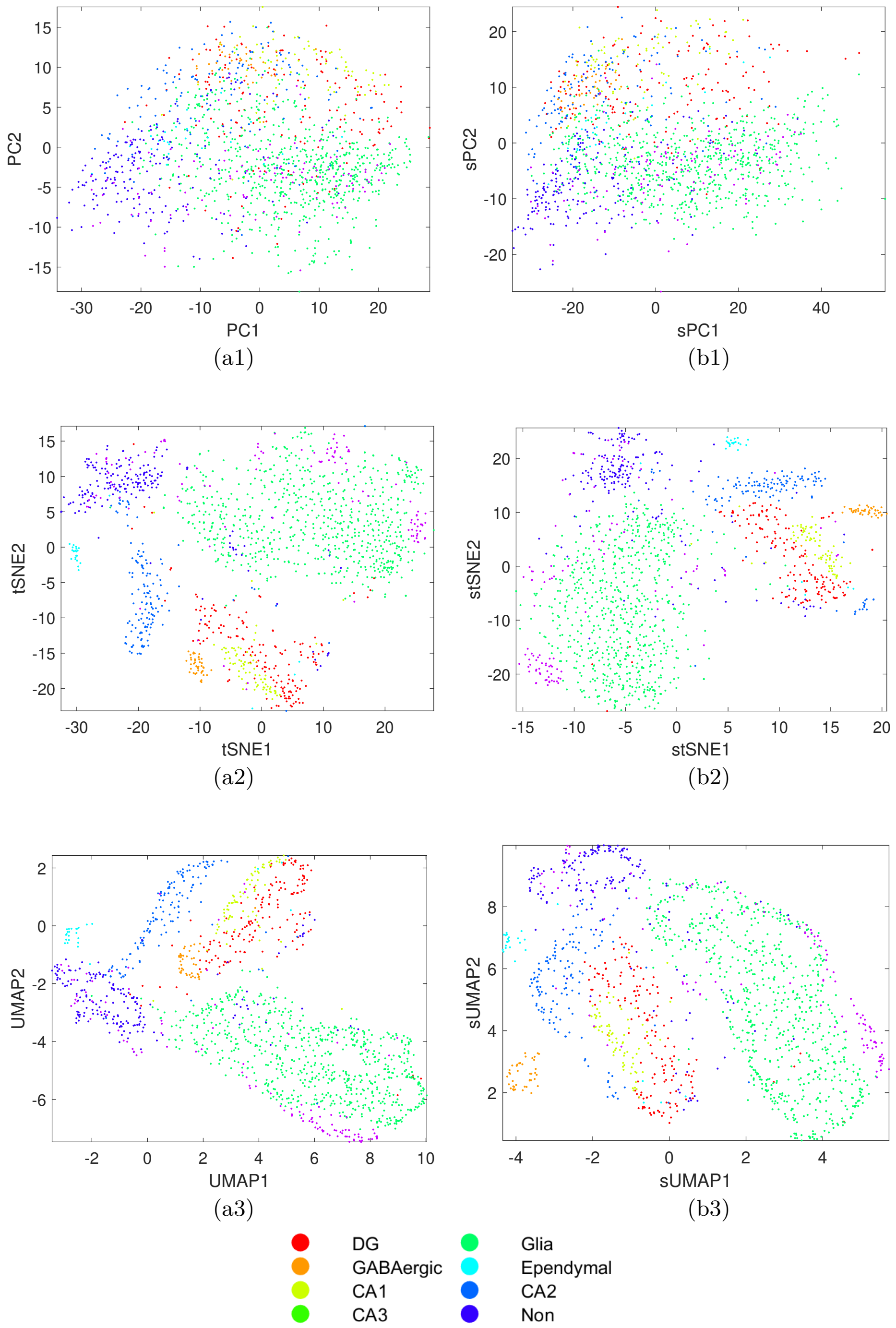

Fig. S17. Div-Seq dataset. (a1,a2,a3) PCA, t-SNE and UMAP results without any imputation. (b1,b2,b3) PCA, t-SNE and UMAP results after SERM imputation.

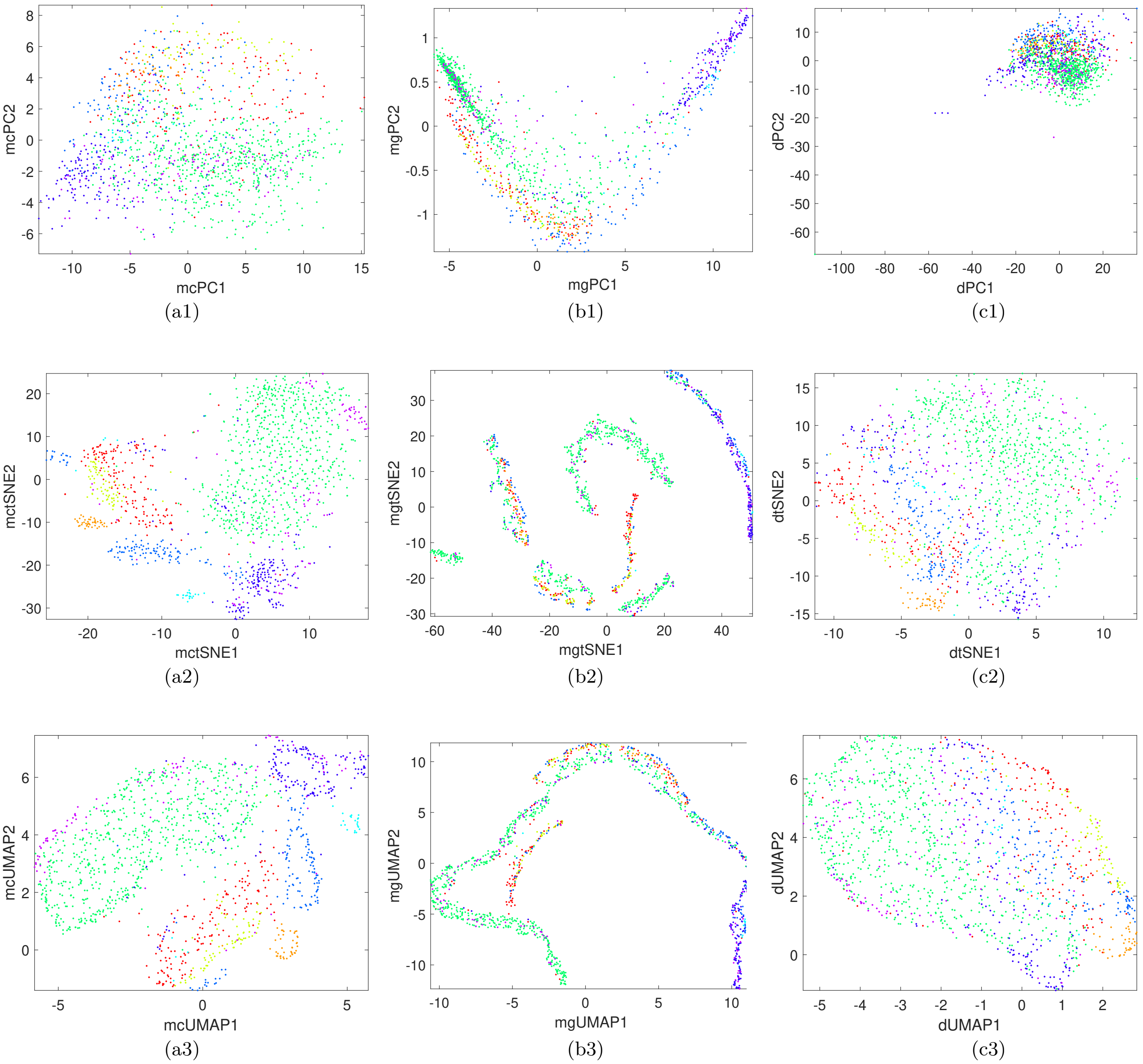

Fig. S18. Div-Seq dataset. (a1,a2,a3) PCA, t-SNE and UMAP results with mcImpute imputation. (b1,b2,b3) PCA, t-SNE and UMAP results after MAGIC imputation. (c1,c2,c3) PCA, t-SNE and UMAP results after DeepImpute imputation. Color legends are shown in Fig. S17.

6. Clustering of intestinal immune cell atlas

This dataset is from a study of signaling abnormalities in immune responses in the small intestine (5). It has been found from this study that signaling abnormalities in immune responses can trigger chronic type 2 inflammation, involving the interaction of multiple immune cell types. To characterize this response systematically, the authors analyzed 58,067 immune cells from the mouse small intestine by scRNA-seq at two states: 1) steady-state and 2) after induction of a type 2 inflammatory reaction to ovalbumin (OVA) (5). The analysis revealed broad shifts in both cell-type composition and cell programs in response to the inflammation, especially in group 2 innate lymphoid cells (ILC2s). The work also highlighted a model where α-CGRP-mediated neuronal signaling is critical for suppressing ILC2 expansion and maintaining homeostasis of type 2 immune machinery.

- Resting CD4+ T cell
- Resting B cell
- ILC3
- LTi cell
- CD8+ T cell
- pDC
- Activated CD4+ T cell
- NK cell
- GC B cell (DZ)
- GC B cell (LZ)
- ILC1
- ILC2
- CD4+ T cell (low UMI count)
- DC (CD103+CD11b+)
- ILC3 (low UMI count)
- LTi cell (low UMI count)
- $\gamma\ \sigma$ T cell (Xcl1+)
- Unresolved
- NKT cell
- Plasma cell
- DC (D103+CD11b−)
- Macrophage
- Resting B cell (low UMI count)
- DC (D103− C1)
- Endothelial cell
- Mast cell
- NK cell (low UMI count)
- GC B cell (low UMI count)
- Doublets
- Epithelial cell C1
- DC (D103− C2)
- Stromal cell (DN)
- Fibroblast
- Unresolved
- Epithelial cell C2
- Neutrophil
- Unresolved
- $\gamma\ \sigma$ T cell (Gzma+)
- Epithelial cell (low UMI count)
- Macrophage (low UMI count)
- Doublets
- Doublets
- Doublets
- Basophil
- T precursor-like cell
- Lymphatic endothelial-like cell

Fig. S19. Legends for cell subsets in the intestinal immune cell atlas shown in Figs. S20 and S21.

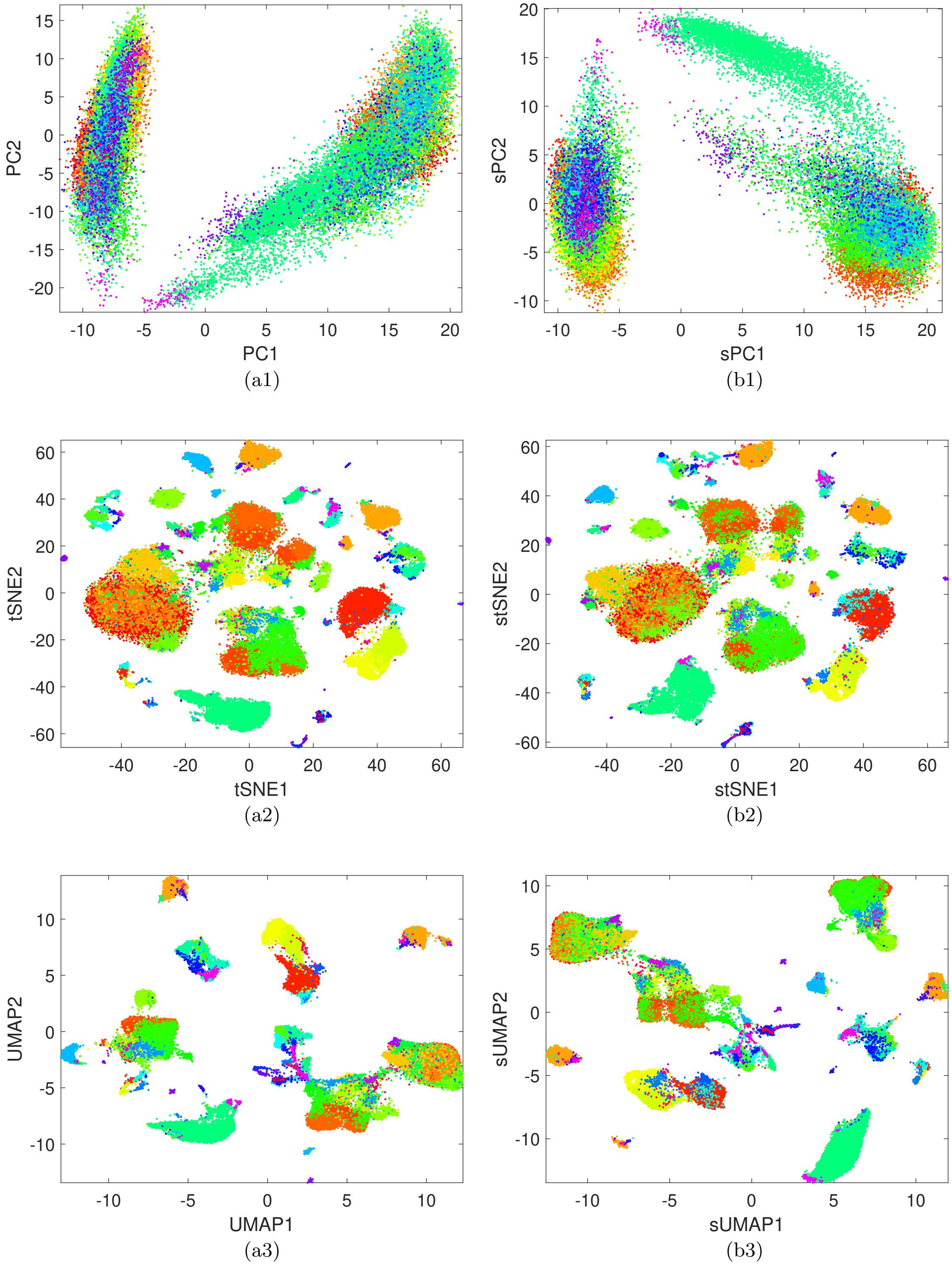

Fig. S20. Intestinal immune cell atlas. (a1,a2,a3) PCA, t-SNE and UMAP results without any imputation. (b1,b2,b3) PCA, t-SNE and UMAP results after SERM imputation.

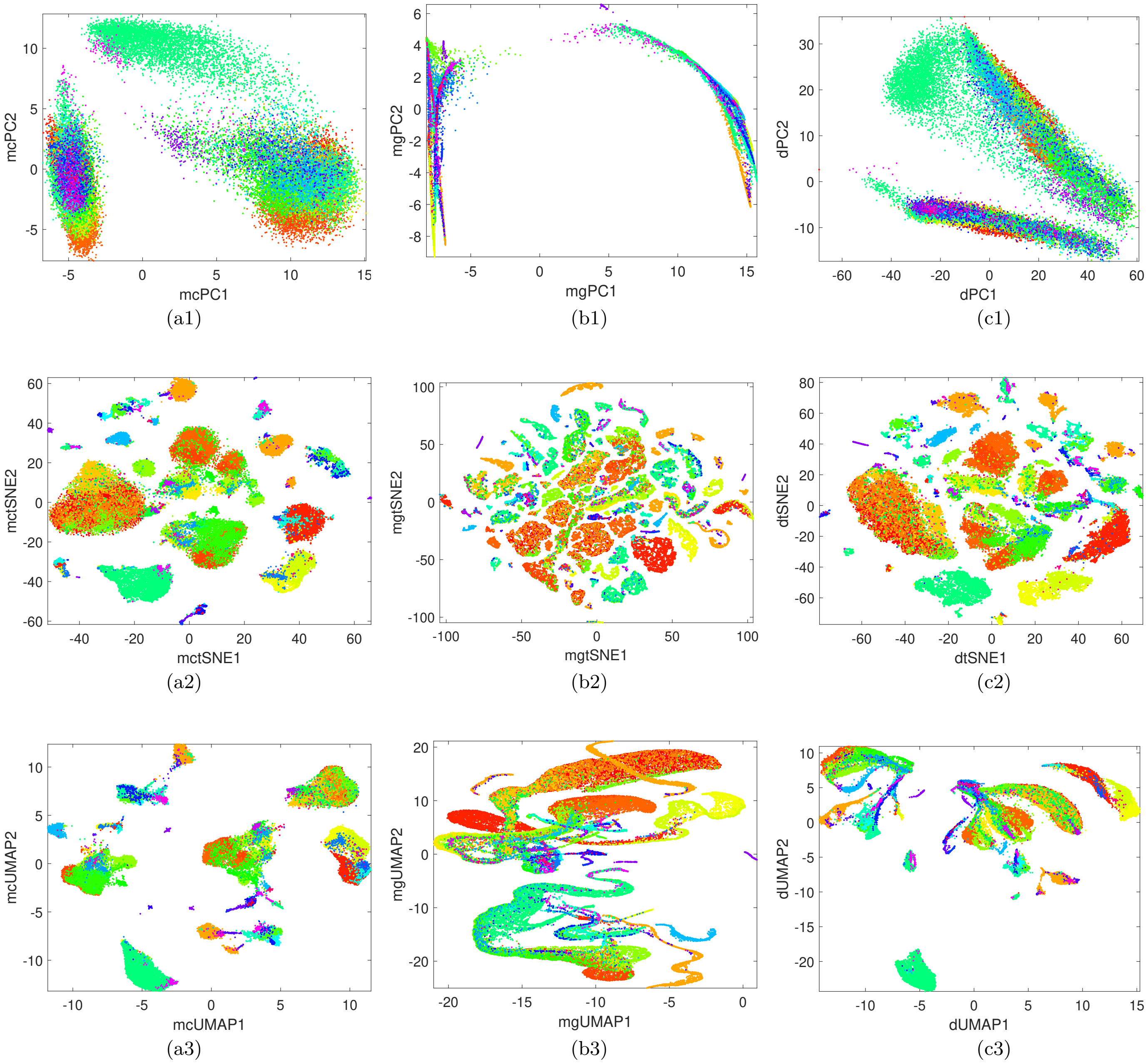

Fig. S21. Intestinal immune cell atlas. (a1,a2,a3) PCA, t-SNE and UMAP results with mcImpute imputation. (b1,b2,b3) PCA, t-SNE and UMAP results after MAGIC imputation. (c1,c2,c3) PCA, t-SNE and UMAP results after DeepImpute imputation.

7. Computational speed

Table 1. Computational speed (in second) of different techniques for different cell number (in thousand) with 1000 genes (shown as bar plot in Fig. S22 (a))

Cell no	mcImpute	MAGIC	AutoImpute	DeepImpute	SAUCIE	DCA	scVI	SERM
10.0	1370.0	194.0	226.0	165.0	62.9	218.0	652.0	50.1
20.0	2390.0	646.0	577.0	471.0	82.3	544.0	1250.0	51.0
30.0	3350.0	1280.0	901.0	947.0	108.0	870.0	1860.0	55.6
40.0	4180.0	2010.0	1210.0	1590.0	139.0	1200.0	2460.0	55.3
50.0	5580.0	2910.0	1570.0	2410.0	176.0	1520.0	3060.0	60.9
60.0	6470.0	4350.0	1850.0	3400.0	219.0	1850.0	3670.0	60.7
70.0	7510.0	5570.0	2220.0	4560.0	267.0	2180.0	4270.0	66.7
80.0	8000.0	6740.0	2510.0	5890.0	322.0	2500.0	4870.0	70.1
90.0	9430.0	8300.0	2860.0	7390.0	382.0	2830.0	5480.0	66.6
100.0	1.03e+4	1.11e+4	3180.0	9060.0	449.0	3160.0	6080.0	70.0

Table 2. Computational speed (in second) of different techniques for different gene number (in thousand) with 10000 cells (shown as bar plot in Fig. S22 (b))

Gene no	mcImpute	MAGIC	AutoImpute	DeepImpute	SAUCIE	DCA	scVI	SERM
2.0	3130.0	156.0	425.0	797.0	108.0	375.0	988.0	51.2
4.0	1.92e+4	182.0	691.0	3610.0	283.0	690.0	1660.0	53.8
6.0	5.1e+4	191.0	1050.0	8480.0	553.0	1000.0	2340.0	57.8
8.0	8.85e+4	182.0	1350.0	1.54e+4	917.0	1320.0	3010.0	61.3
10.0	1.32e+5	190.0	1660.0	2.44e+4	1380.0	1630.0	3680.0	65.8
12.0	1.54e+5	201.0	1960.0	3.55e+4	1930.0	1950.0	4360.0	68.5
14.0	1.78e+5	212.0	2270.0	4.86e+4	2580.0	2260.0	5030.0	73.2
16.0	1.71e+5	218.0	2630.0	6.38e+4	3320.0	2580.0	5700.0	77.4
18.0	1.81e+5	208.0	2900.0	8.1e+4	4160.0	2890.0	6380.0	79.9
20.0	1.87e+5	211.0	3240.0	1.0e+5	5090.0	3210.0	7050.0	83.5

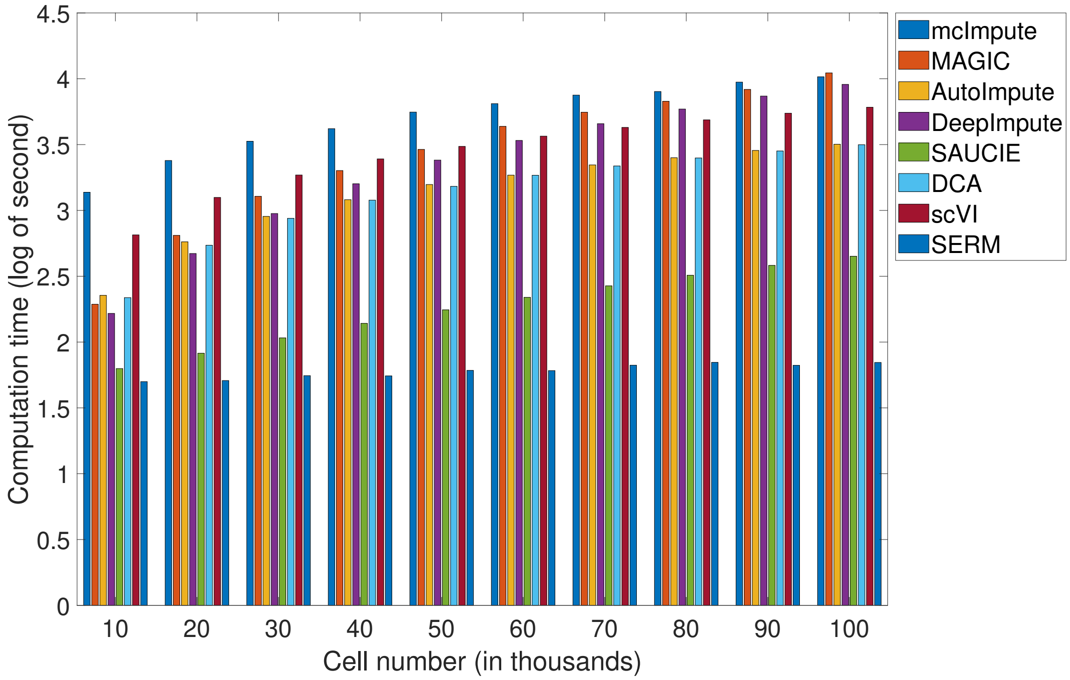

(a)

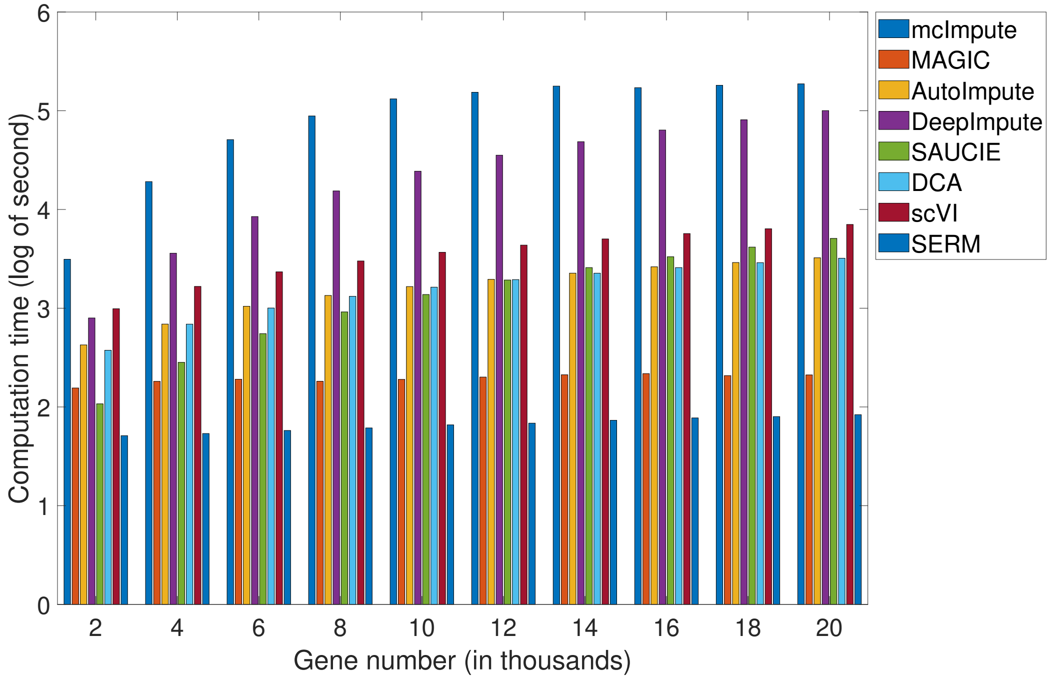

(b)

Fig. S22. Computational speed of eight different techniques for (a) different number of cells with 1000 genes (b) different number of genes with 10,000 cells.

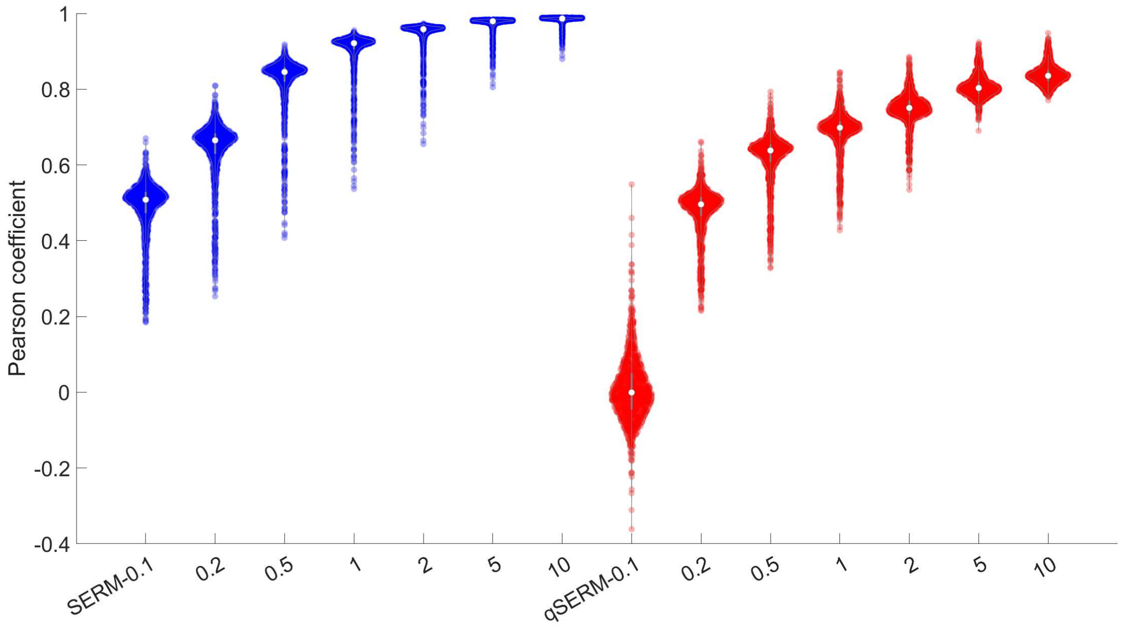

Fig. S23. Pearson correlation values for SERM with adaptive histogram equalization (SERM) and quantile normalization (qSERM) for cellular taxonomy dataset for different sampling efficiencies (%).

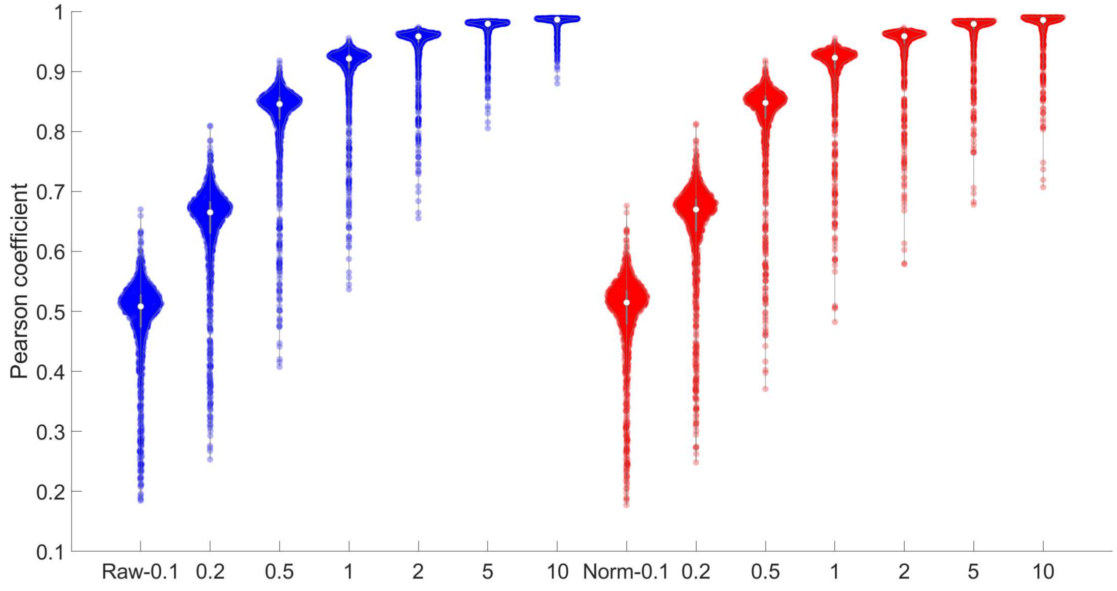

Fig. S24. Pearson correlation values of SERM-imputed data for the raw and library normalized data for cellular taxonomy dataset for different sampling efficiencies (%).

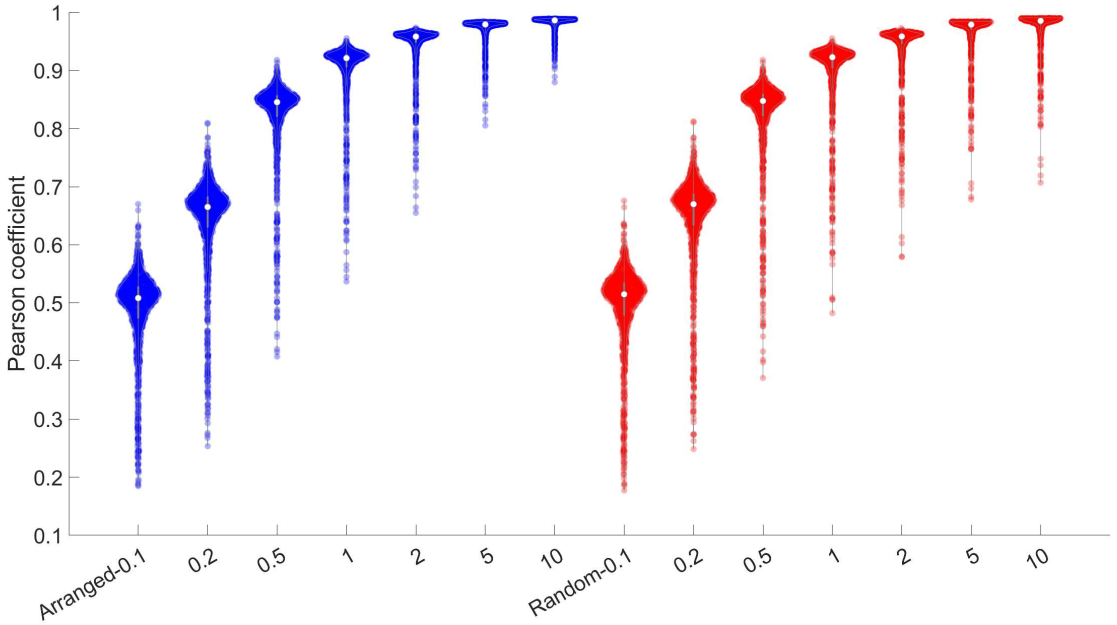

Fig. S25. Pearson correlation values of SERM-imputed data for random and arranged data based on cell type for cellular taxonomy dataset for different sampling efficiencies (%).

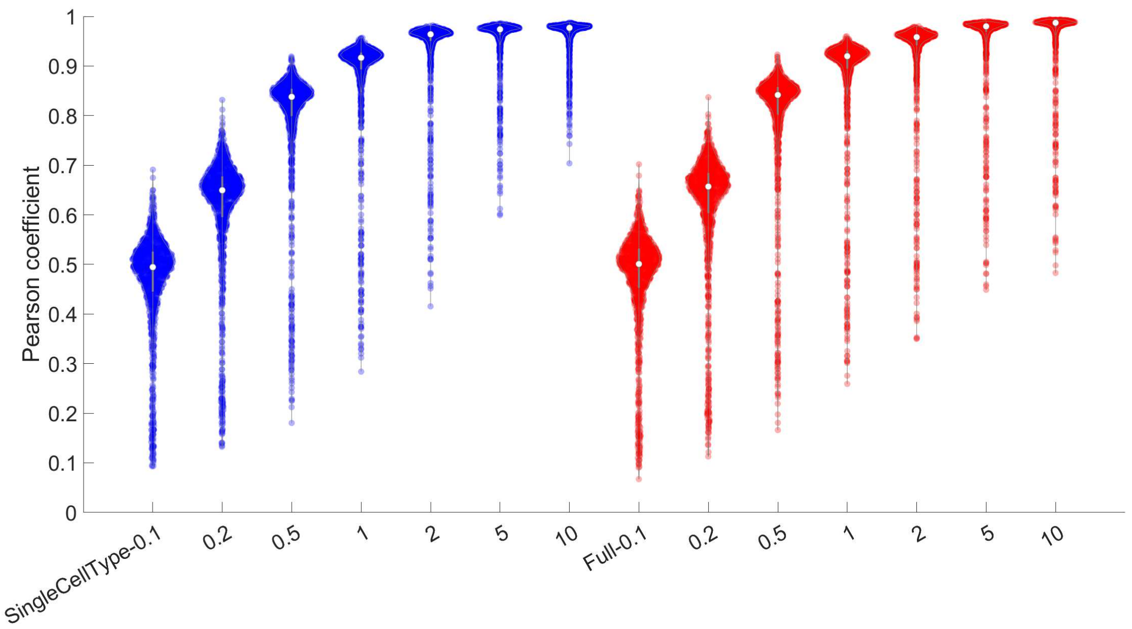

Fig. S26. Pearson correlation values for SERM imputation for fractional data (only one cell type-EC sinusoidal) and full data of cellular taxonomy dataset for different sampling efficiencies (%).

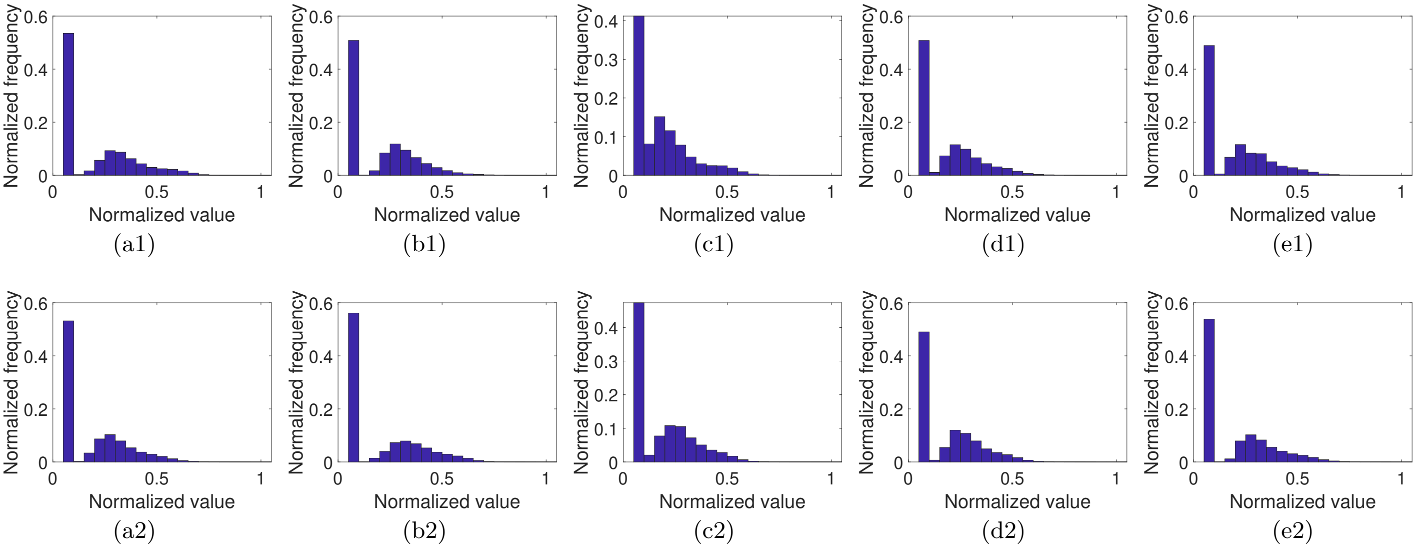

Fig. S27. Distribution of gene expressions in 10 different cell types of cellular taxonomy dataset. The distributions for EC-sinusoidal, MSC, Chondro-hyper, Fibro-4, and Chondro-progen cells are shown in (a1-e1), and for Fibro-5, EC-arterial, OLC-1, OLC-2, and Fibro-1 cells are shown in (a2-e2), respectively.

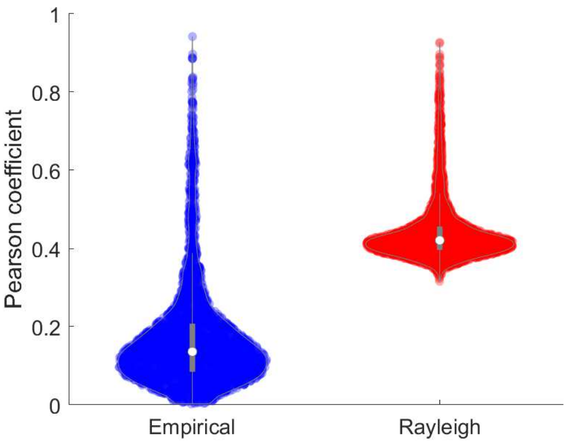

Fig. S28. Pearson correlation values for the empirical and Rayleigh distribution in SERM for 3D neural tissue dataset.

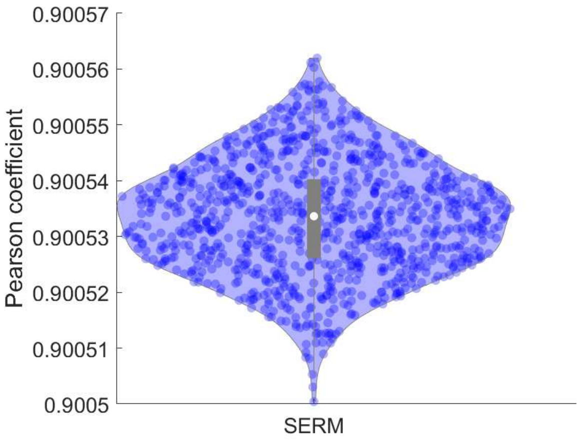

Fig. S29. Distribution of Pearson correlation values for 1000 randomization of data points in SERM for observed data (sampled at 5% efficiency) of 3D neural tissue dataset.

8. Distribution of denoised data learned when different number of data points are used

We used the reference and sampled 3D neural tissue dataset at 1% efficiency for this analyses. The dataset consists of 2,364 cells and 2,735 genes. We used different number of data points (50-2350 at step of 50) randomly for learning the distribution of auto-encoder reconstructed data. The learned distribution is listed in Table 3. The Pearson correlation coefficient between the imputed data and reference data is shown in Fig. S30 for different learned disributions. The parameter distribution of the learned distributions are shown in Figs. S31 and S32.

Table 3. Learned distribution when different number of points are used in case of 3D neural dataset.

Learned distribution	Data points used in the learning
Rayleigh	50-350, 500, 550-700,800,900,1200
Exponential	400,450,750,850, 950-1100,1250-2350

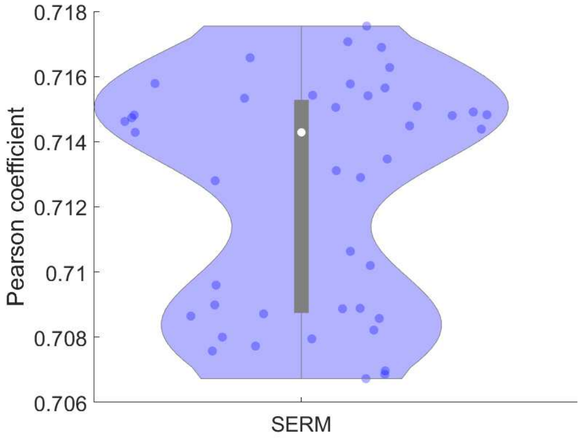

Fig. S30. Distribution of Pearson correlation values for 47 different size of the data used in learning the data distribution in SERM.

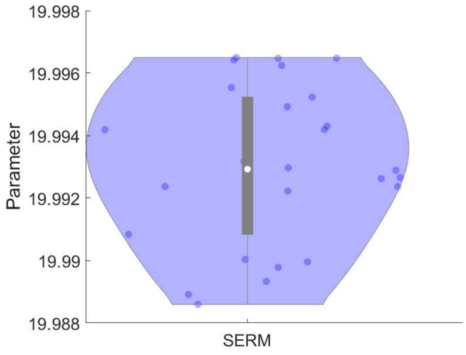

Fig. S31. Distribution of parameter values when exponential distribution is selected in SERM.

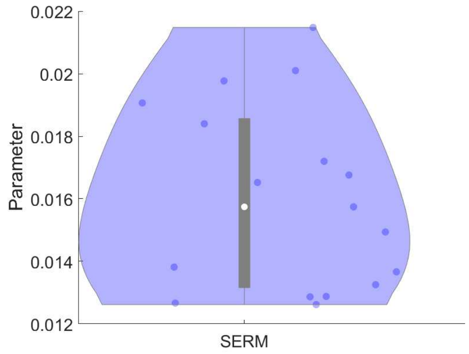

Fig. S32. Distribution of parameter values when Rayleigh distribution is selected in SERM.

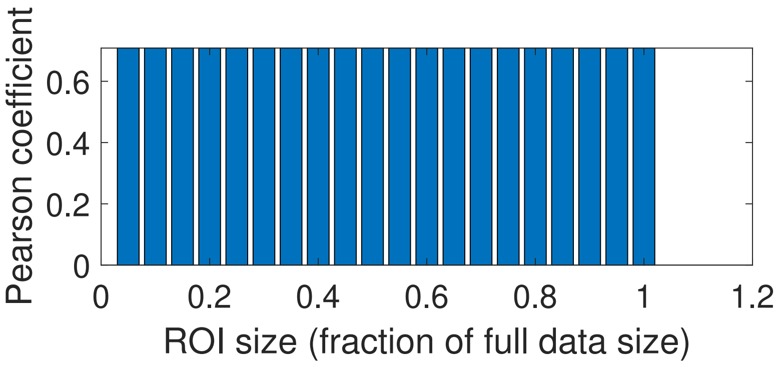

Fig. S33. Distribution of Pearson coefficients for different ROI sizes in SERM for 3D neural tissue dataset.

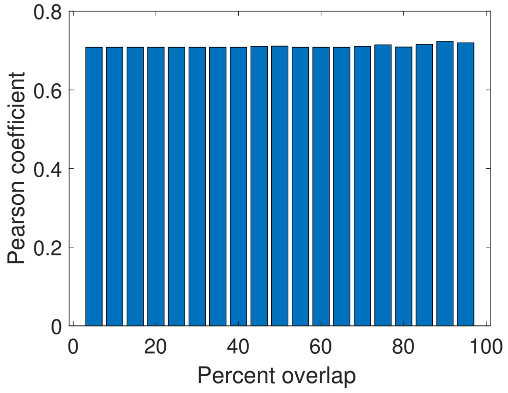

Fig. S34. Distribution of Pearson coefficients for different percent overlaps in SERM for 3D neural tissue dataset.

9. HCL and MCA datasets

The t-SNE visualization of the HCL and MCA datasets without imputation is shown in Fig. S35, whereas the t-SNE visualization of the SERM-imputed data is shown in Fig. S36. From these figures, it is seen that the data classes are reliably clustered with SERM imputation. The results of DeepImpute and SAUCIE are shown in Figs. S37 and S38. The quantitative performance of different techniques in terms of clustering accuracy and cluster quality indices are shown in Fig. S39.

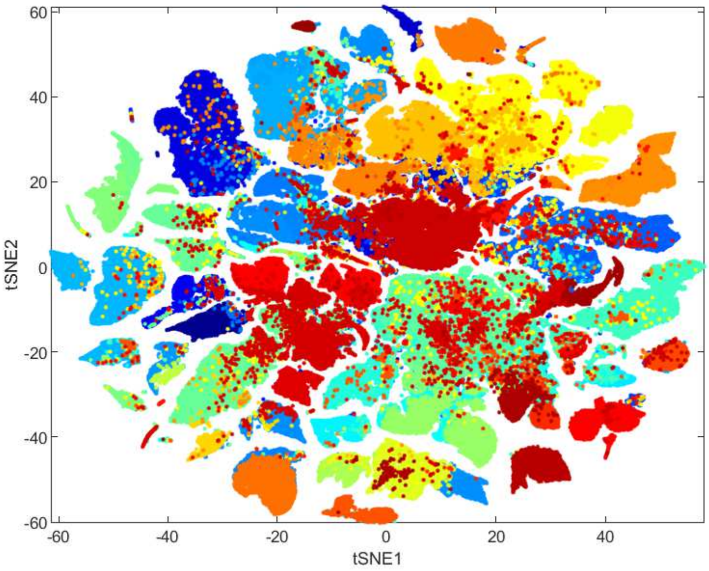

(a)

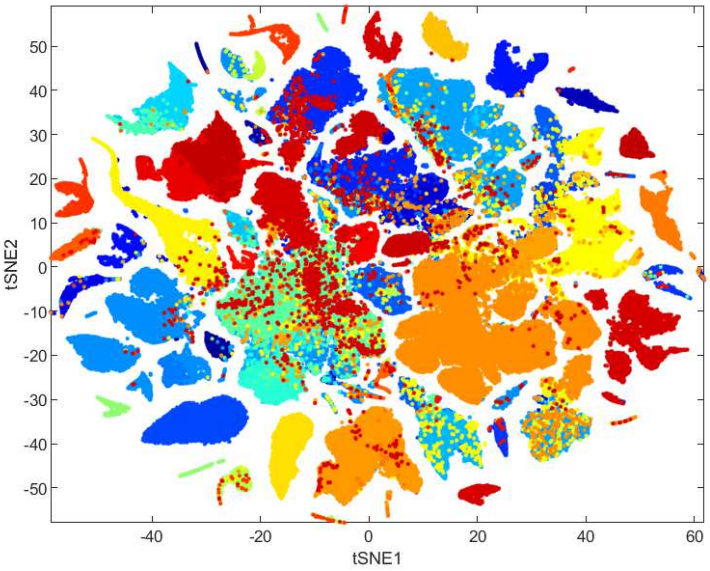

(b)

Fig. S35. t-SNE visualization of human cell landscape (a) and mouse cell atlas (b) dataset without imputation.

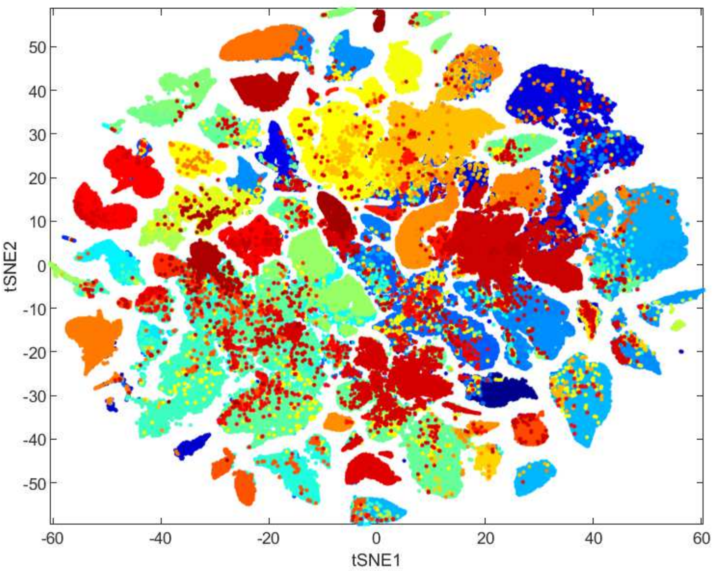

(a)

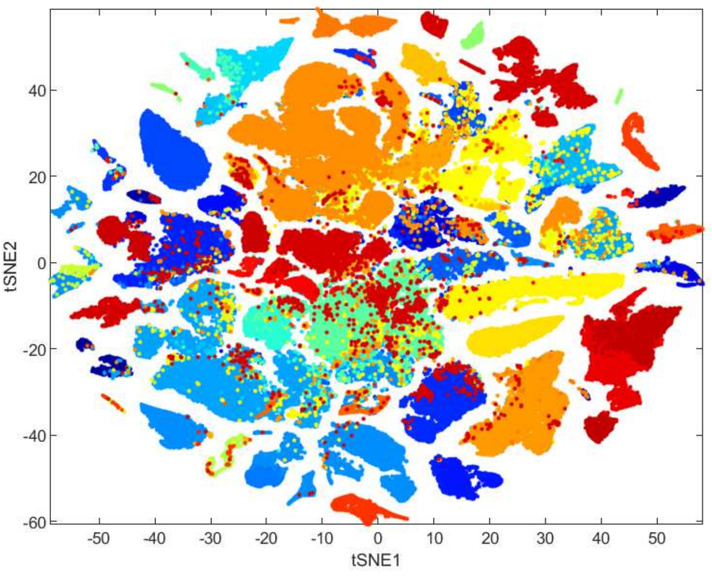

(b)

Fig. S36. t-SNE visualization of human cell landscape (a) and mouse cell atlas (b) dataset imputed by SERM.

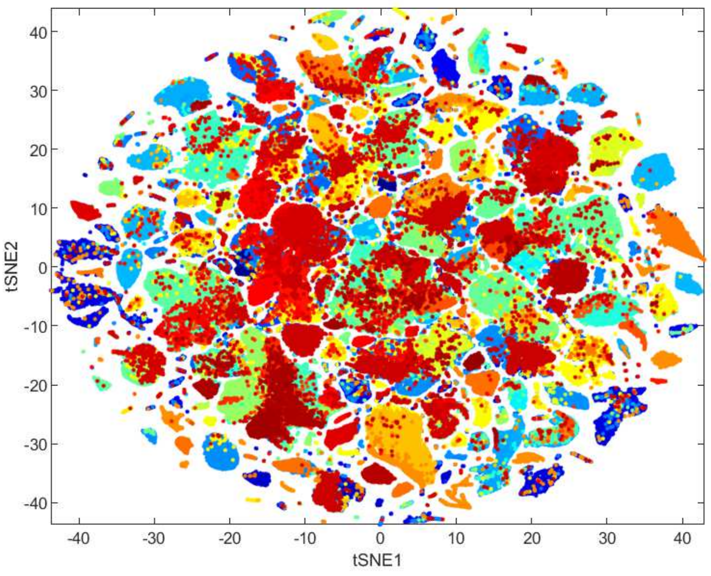

(a)

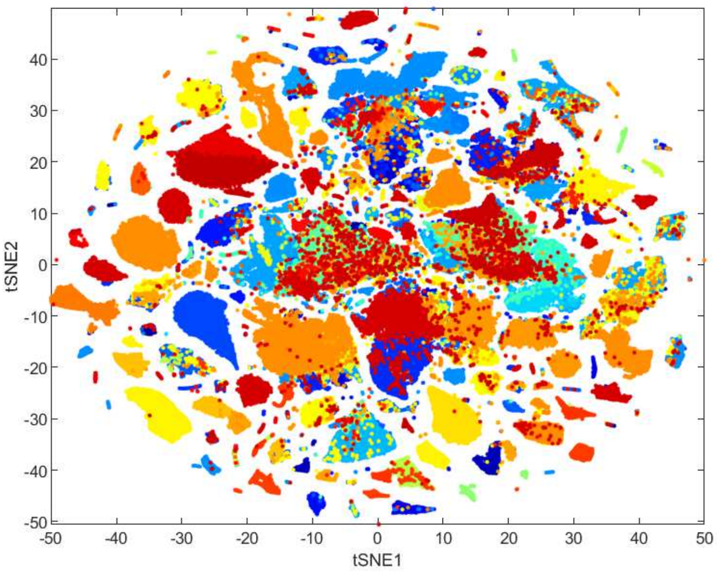

(b)

Fig. S37. t-SNE visualization of human cell landscape (a) and mouse cell atlas (b) dataset imputed by DeepImpute.

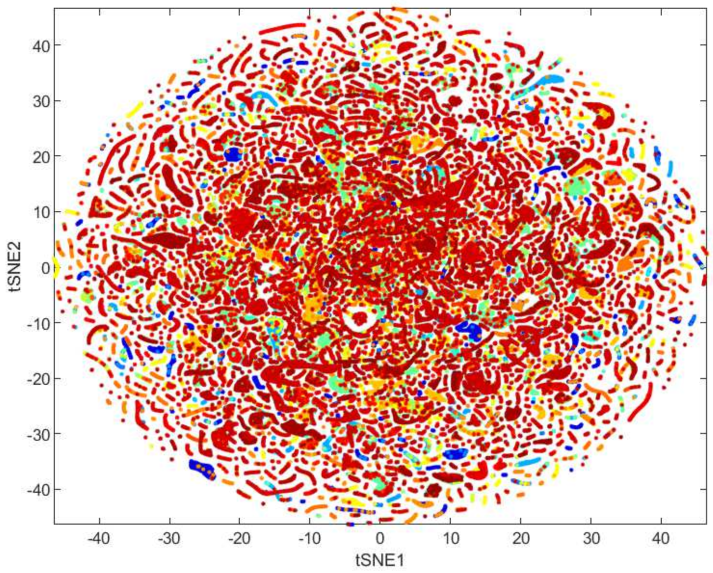

(a)

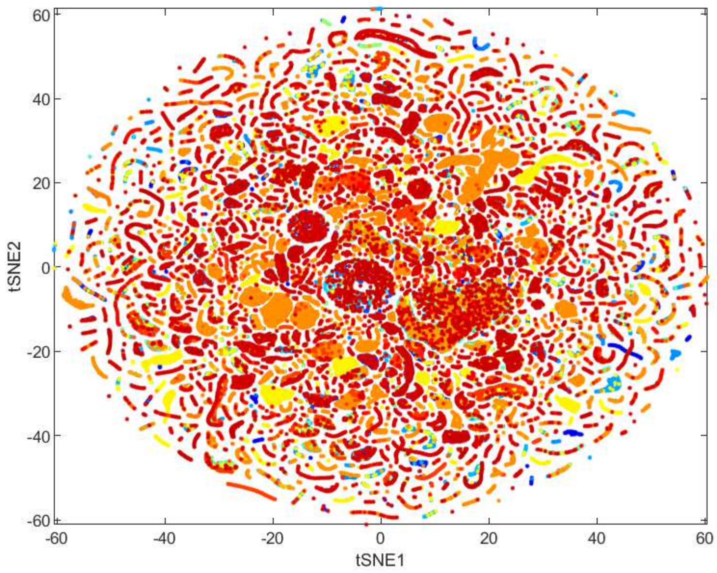

(b)

Fig. S38. t-SNE visualization of human cell landscape (a) and mouse cell atlas (b) dataset imputed by SAUCIE.

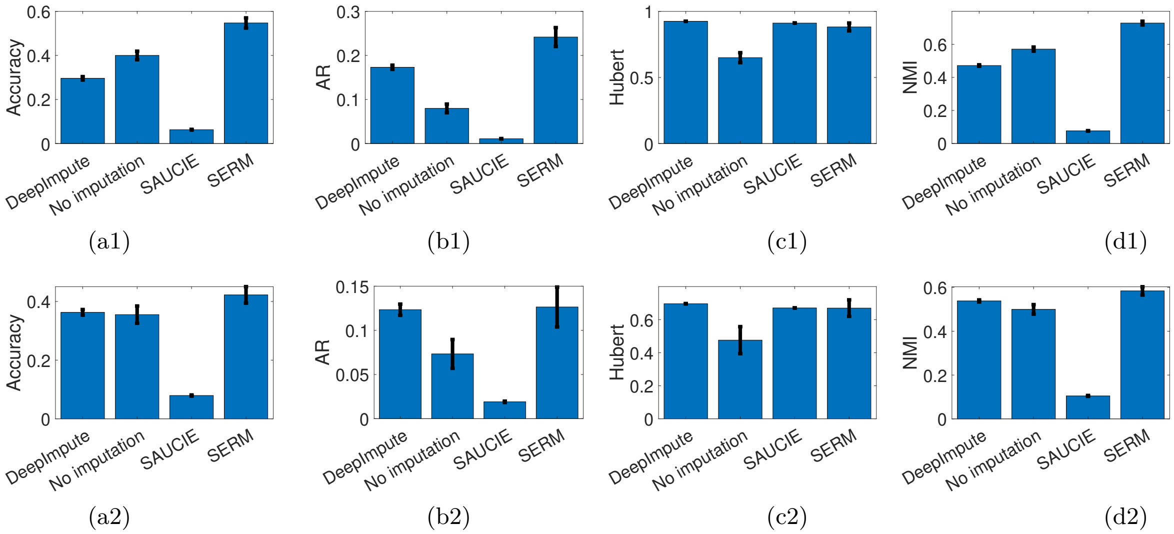

Fig. S39. Accuracy (a), AR (b), Hubert (c) and NMI (d) indices of unimputed and imputed data from DeepImpute, SAUCIE, and SERM for human cell landscape (1) and mouse cell atlas (2) datasets. Error bars represent the standard deviation of the indices for 1000 different initializations of k-means clustering.

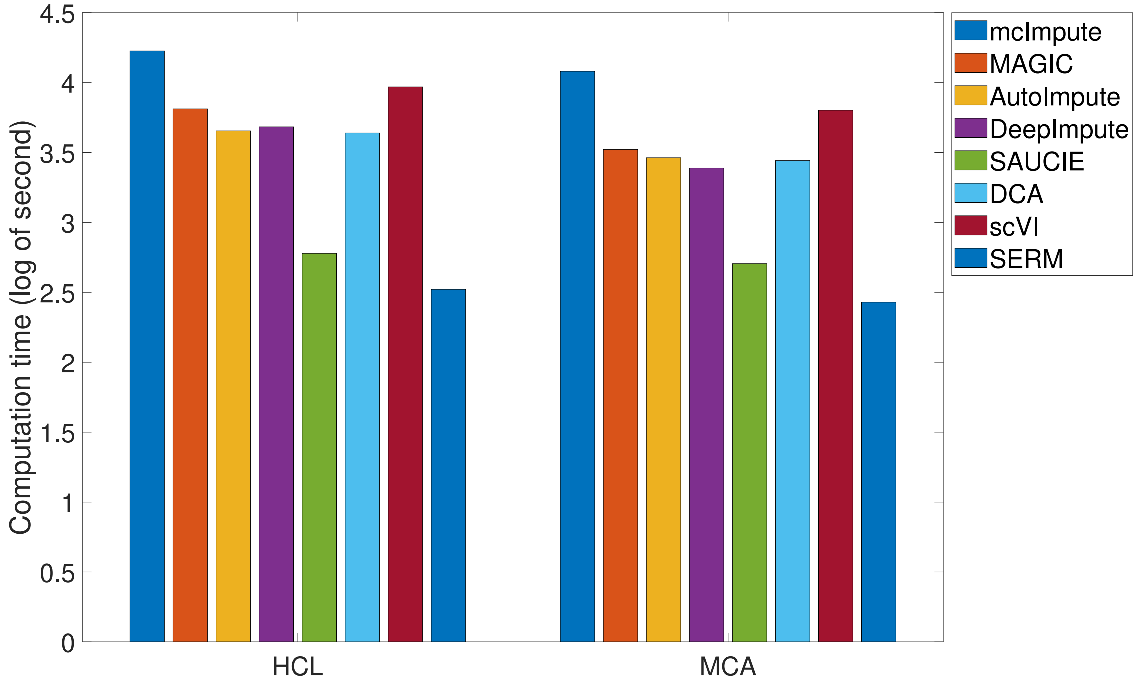

Fig. S40. Computational speed of different techniques for imputing subsampled (by a factor of 20) HCL and MCA datasets.

10. Slingshot and TSCAN analysis of imputed data from different methods

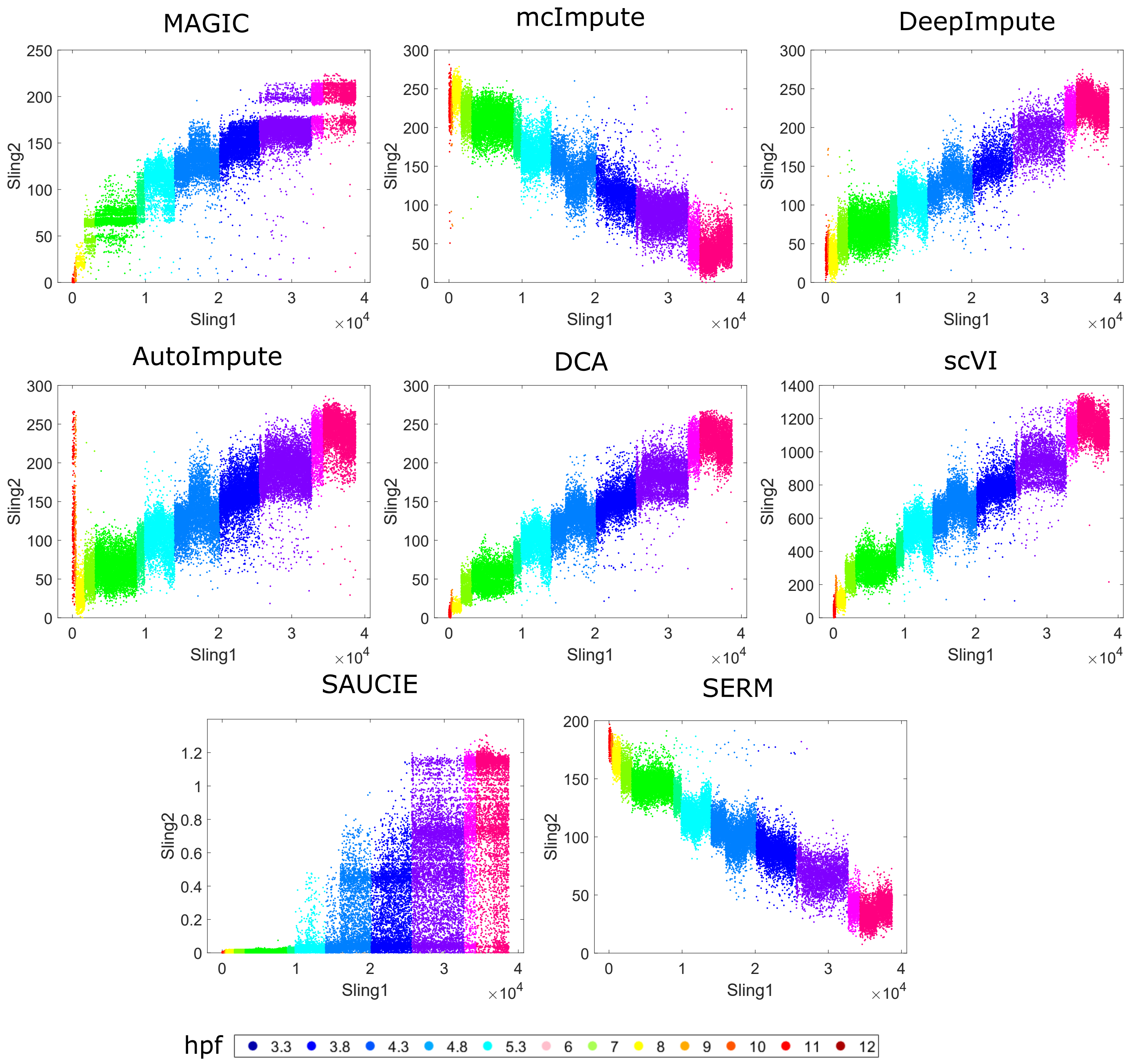

Fig. S41. Slingshot visualizations of the imputed data by eight different methods for the zebrafish development data.

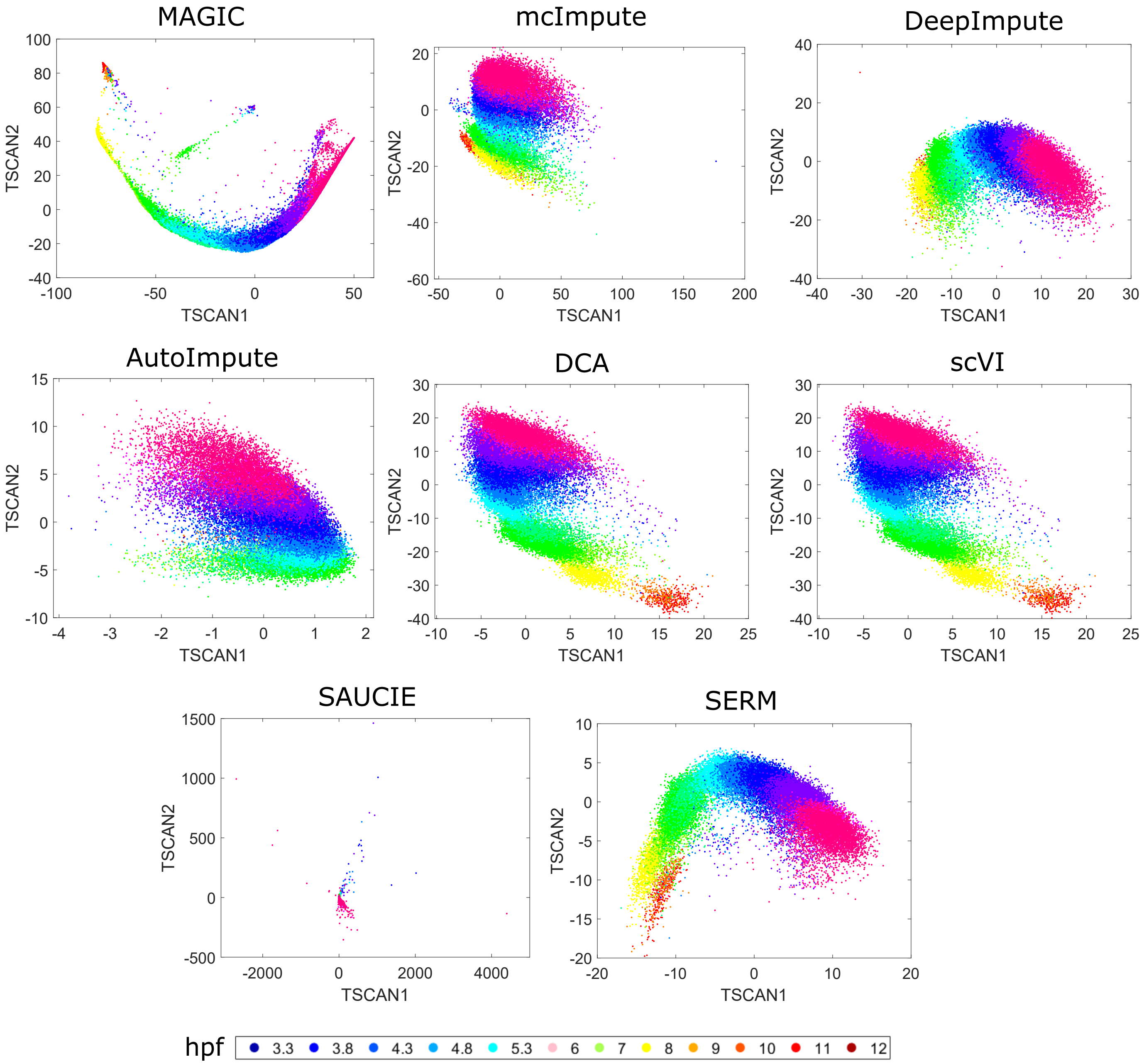

Fig. S42. TSCAN visualizations of the imputed data by eight different methods for the zebrafish development data.

11. Batch effect correction by SERM

To demonstrate the usage of SERM in batch-effect correction, we choose five human pancreatic datasets from the works of Segerstolpe et al. (6), Baron et al. (7), Muraro et al. (8), Xin et al. (9), and Wang et al (10). The datasets were acquired using SMART- Seq2 , inDrop, CEL-Seq2, and SMARTer scRNA-seq technologies. The raw data from Baron et al., Muraro et al., Segerstolpe et al., Xin et al., and Wang et al. contained 8,569, 2,122, 2,127, 1,492, and 457 cells. All the five datasets had 15,558 genes. We selected the most 2,000 variable genes from Baron dataset and chose only the expression values of those genes from all other datasets. It lead to 8,569, 2,122, 2,127, 1,492, and 457 cells from the five datasets with 2,000 genes. We next combined the five datasets into a single dataset and different imputation methods were applied to impute the expression values of the combined dataset. The calculation process of SERM for such data with batch-effect is shown in Fig. S43. The comparison of performance of different imputation methods in batch-effect correction is shown in Figs. S44, S45 and S46.

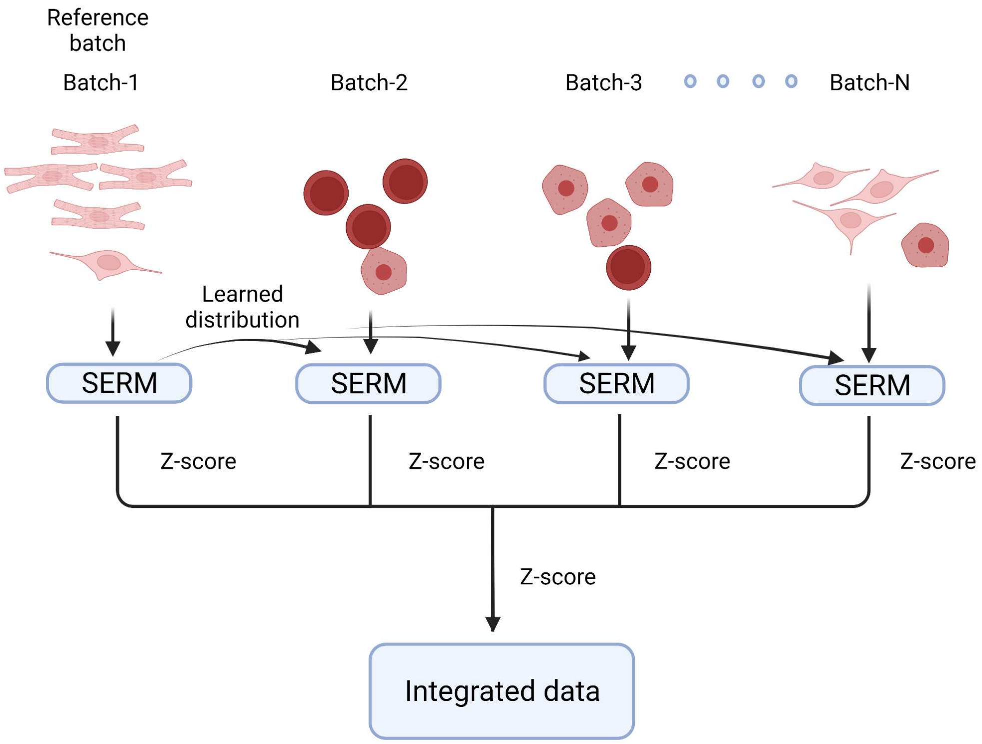

Fig. S43. Workflow for batch-effect correction using SERM. Here, we show batch 1 as the reference batch. After the distribution learning, SERM imputes all the batches using the distribution. We note that any of the batches can be selected as the reference and there is little change in the performance of SERM when a different reference dataset is used.

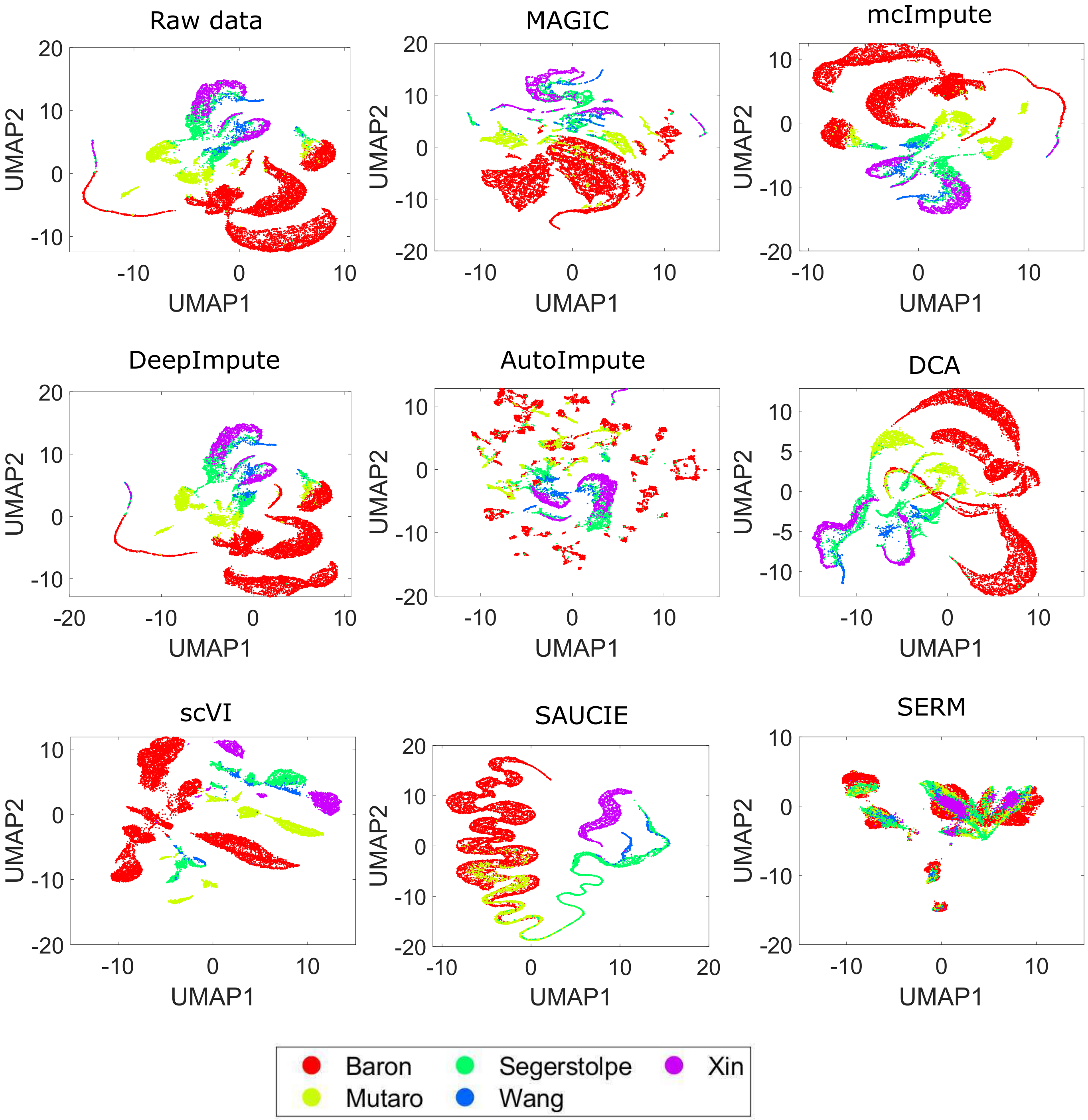

Fig. S44. Comparison of different imputation methods for batch effect correction on the human pancreas dataset. UMAP visualizations of the raw and imputed data by eight different methods for HD datasets obtained by using five different measurement protocols. Data of different measurement protocols are denoted by different colors.

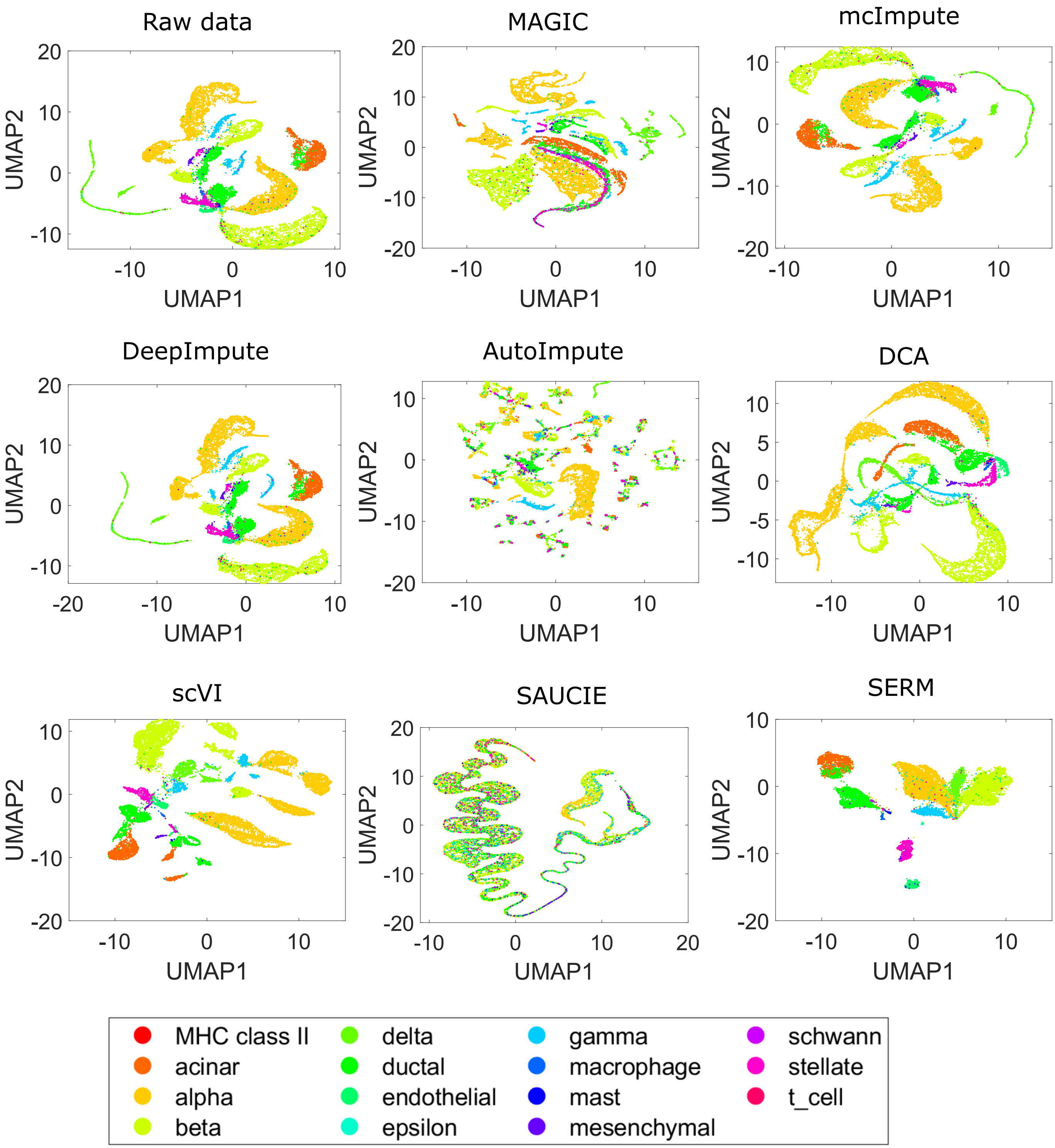

Fig. S45. Comparison of different imputation methods for batch effect correction on the human pancreas dataset. UMAP visualizations of the raw and imputed data by eight different methods for HD datasets obtained by using five different measurement protocols. Data of different cell types are denoted by different colors.

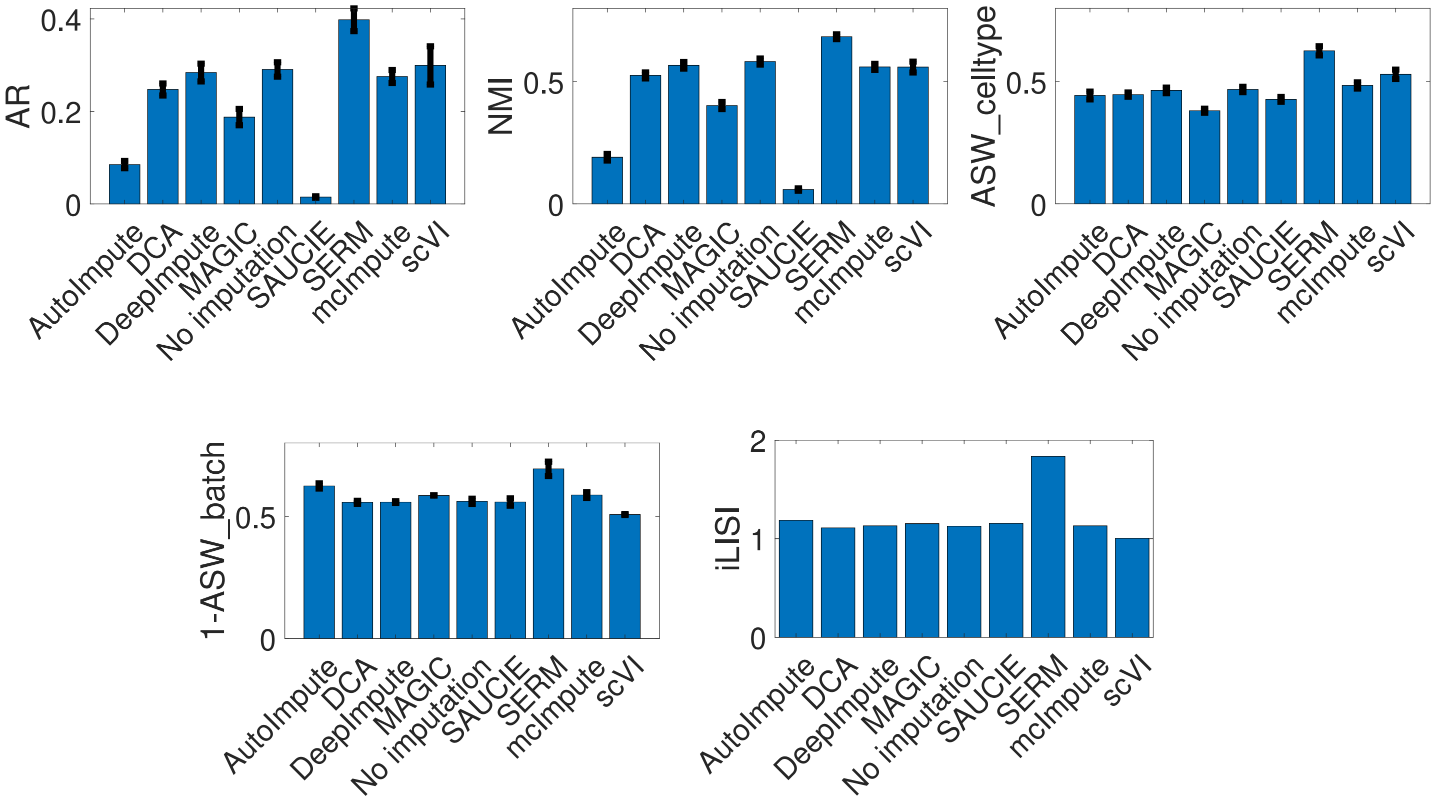

Fig. S46. Comparison of different imputation methods for batch effect correction on the human pancreas dataset. Bar plots of metrics ARI, NMI, ASW celltype, 1-ASW_batch, and iLISI for the 8 methods, with the raw data ('no imputation') as comparison. Higher ARI, NMI and ASW_celltype, indicate better performance in clustering, and higher 1-ASW_batch and iLISI indicate better performance in batch mixing. For calculation methods and codes of the indices, please see Refs. (11), (12), and (13).

12. Statistics of reference and observed datasets

The cellular taxonomy dataset contained 27,998 genes and 23,092 cells. Genes with mean expression value less than 0.001 and nonzero expression in less than three cells were filtered out. The filtered dataset contained 7,703 genes and 23,092 cells. We then selected genes that had nonzero expression in 10% of the cells. Next, we selected the cells that have library size greater than 387,500. We ended up having 2,422 genes and 12,162 cells for the study.

The mammalian brain dataset contained 30,341 genes and 18,194 cells. There was no gene with mean expression of 0.001 and nonzero expression in fewer than three cells. We selected genes that had nonzero expression in 20% of the cells. Then, we selected the cells that have library size greater than 700,000. We ended up having 2,344 genes and 10,360 cells.

The mouse intestinal epithelium dataset contained 15,971 genes and 7,216 cells. Genes with mean expression value less than 0.001 and nonzero expression value in less than three cells were filtered out. The filtered dataset contained 15,252 genes and 7,216 cells. The genes with nonzero expression in 30% of the cells were selected (resulting in 1,776 genes). The reference dataset was built by choosing cells with greater than 760,000 library size, which lead to 4,072 cells and 1,776 genes.

The human engineered neural cells dataset contained 22,567 genes and 4,280 cells. Genes with mean expression value less than 0.001 and nonzero expression value in less than three cells were filtered out. The filtered dataset contained 20,517 genes and 4,280 cells. We then selected genes that had nonzero expression in 30% of the cells, resulting in 2,735 genes. Next, we selected the cells that have library size greater than 820,000. We ended up having 2,735 genes and 2,364 cells.

The Zebrafish embryogenesis dataset contained 17,239 genes and 38,731 cells. There was no gene with mean expression of 0.001 and nonzero expression in fewer than three cells. We then selected genes that had nonzero expression in 20% of the cells, resulting in 2,341 genes. Next, we selected the cells that have library size greater than 880,000. We ended up having 2,341 genes and 20,014 cells.

The EB differentiation dataset contained 17,580 genes and 16,825 cells. Genes with mean expression value less than 0.001 and nonzero expression value in less than three cells were filtered out. The filtered dataset contained 17,577 genes and 16,825 cells. We then selected genes that had nonzero expression in 20% of the cells, resulting in 2,282 genes. Next, we selected the cells that have library size greater than 855,000. We ended up having 2,282 genes and 9,754 cells.

Table 4. Number of cells and genes of analyzed datasets

Dataset	Raw	Reference	Observed
Cellular taxonomy	27,998 genes and 23,092 cells	2,422 genes and 12,162 cells	2,422 genes and 12,162 cells
Mammalian brain	30,341 genes and 18,194 cells	2,344 genes and 10,360 cells	2,344 genes and 10,360 cells
Mouse intestinal epithelium	15,971 genes and 7,216 cells	4,072 cells and 1,776 genes	4,072 cells and 1,776 genes
3D neural tissue data	22,567 genes and 4,280 cells	2,735 genes and 2,364 cells	2,735 genes and 2,364 cells
Zebrafish development	17,239 genes and 38,731 cells	2,341 genes and 20,014 cells	2,341 genes and 20,014 cells
EB differentiation	17,580 genes and 16,825 cells	2,282 genes and 9,754 cells	2,282 genes and 9,754 cells

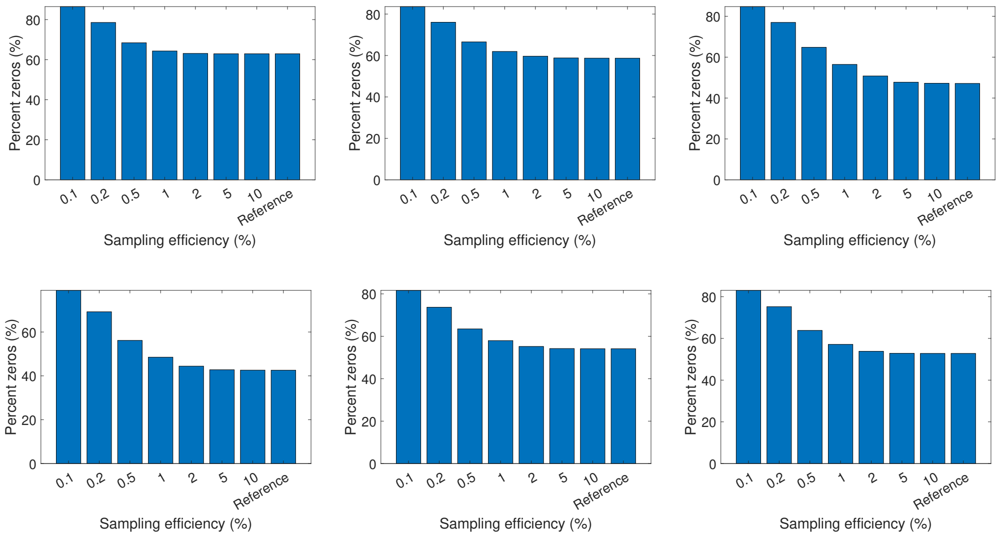

Fig. S47. Percent zeros at different sampling efficiencies to create the observed data for different datasets: (1st row-from left to right) cellular taxonomy, mammalian brain, and 3D neural tissue datasets (2nd row-from left to right) mouse intestinal epithelium, zebrafish development and EB differentiation datasets.

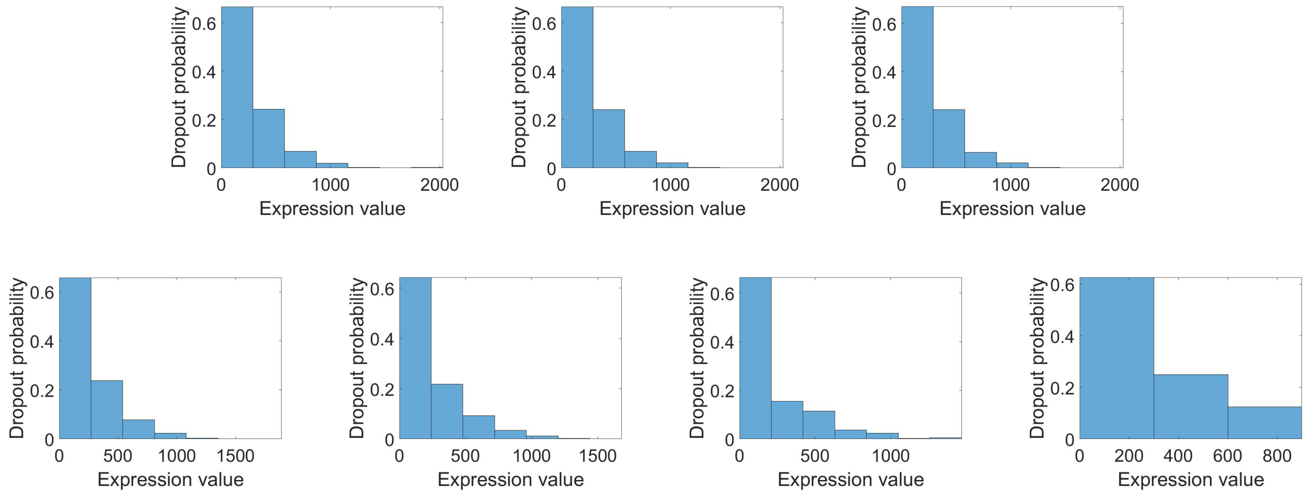

Fig. S48. Dropout probability of the gene expression values at different sampling efficiencies for cellular taxonomy dataset: (1st row-from left to right) 0.1%, 0.2%, and 0.5%, (2nd row-from left to right) 1%, 2%, 5%, and 10% efficiency.

13. Analysis of Tabula Muris (TM) dataset

This dataset represents a compendium of single-cell transcriptomic data from the model organism Mus musculus that comprises cells from 20 organs and tissues of mice (14). The dataset reveals the gene expression in poorly characterized cell populations and enables a direct and controlled comparison of gene expression levels in cell types shared between tissues, such as T lymphocytes and endothelial cells from different anatomical locations (14). The raw data contained 54,765 cells and 19,791 genes. We imputed the data using all the methods and present the results below.

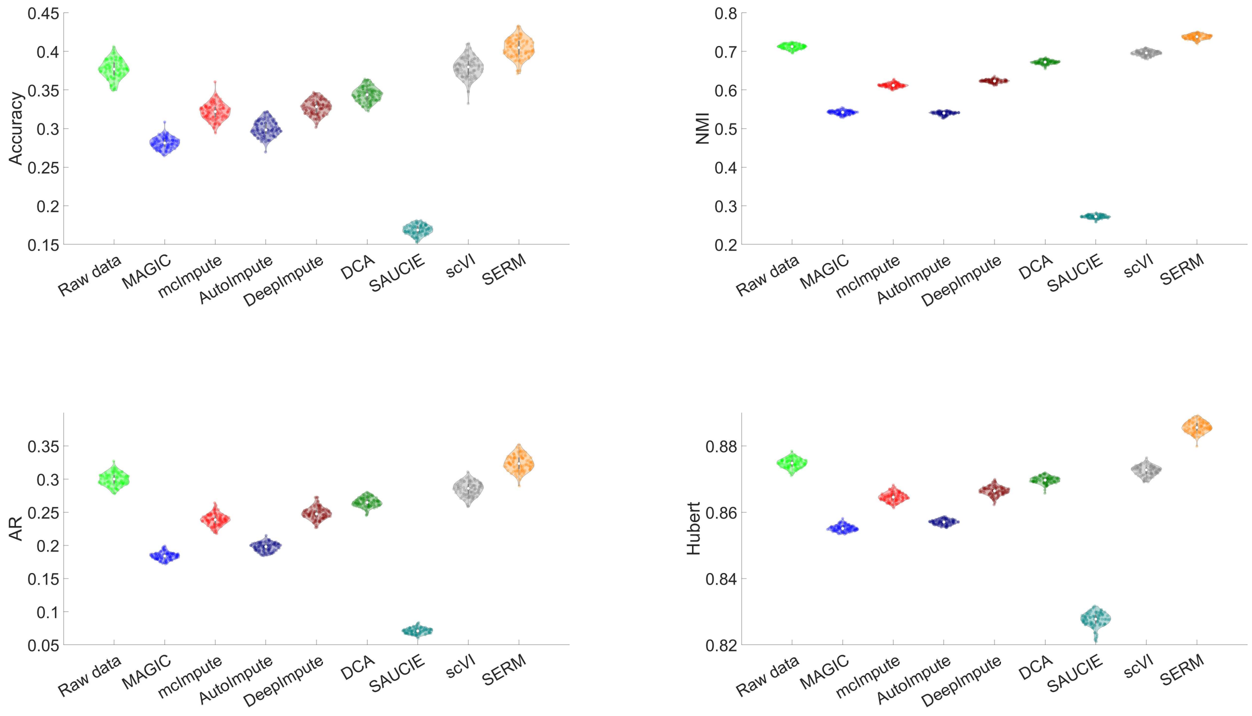

Fig. S49. Comparison of different imputation methods for imputing the TM dataset. Accuracy, NMI, AR, and Hubert indices computed from the t-SNE visualizations (shown in Fig. S50) of TM dataset imputed by different techniques.

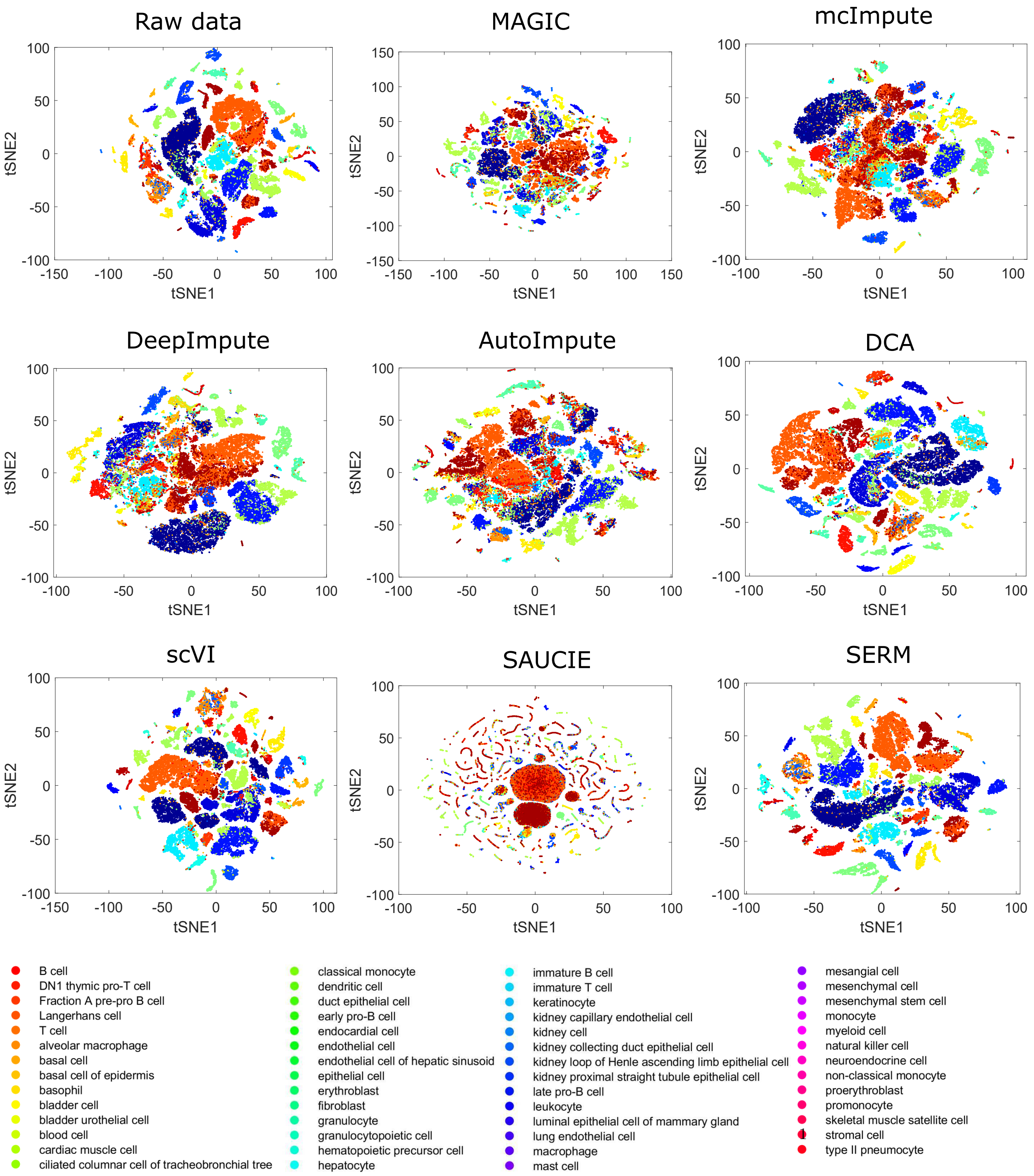

Fig. S50. Comparison of different imputation methods for imputing the TM dataset. t-SNE visualizations of the raw and imputed data by eight different methods. Data of different cell types are denoted by different colors.

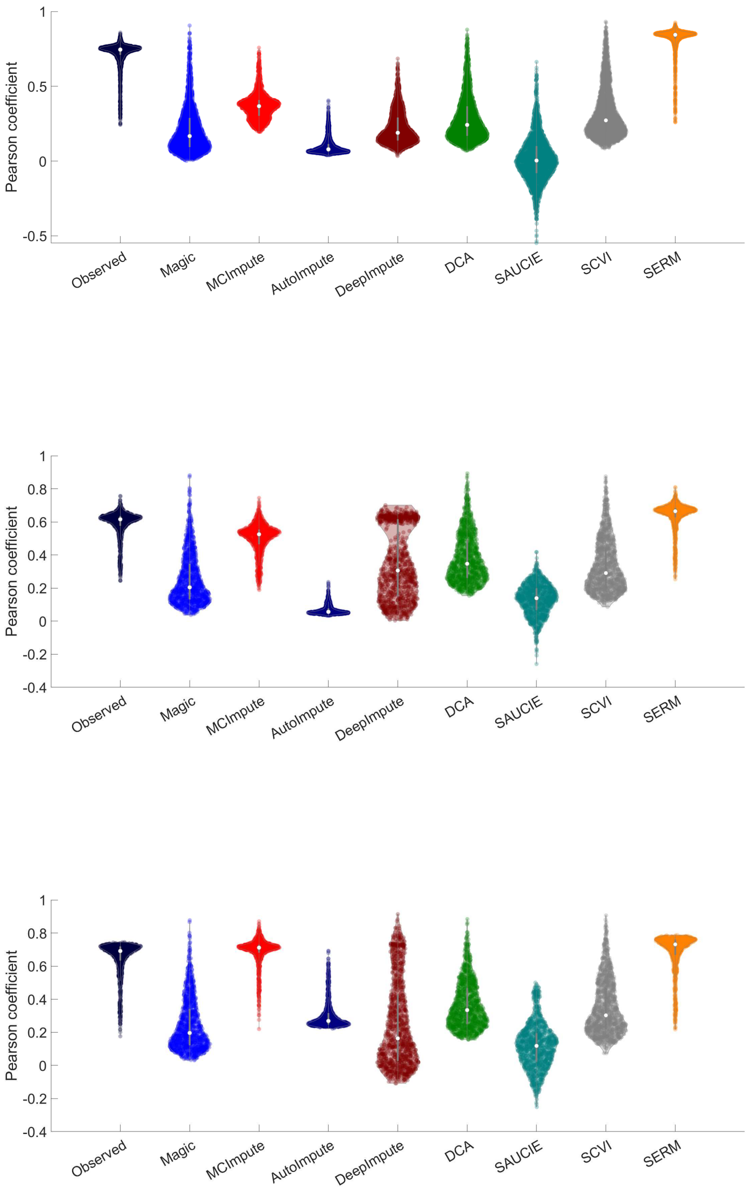

Fig. S51. Pearson coefficient between the reference and imputed data by eight different techniques for cellular taxonomy dataset. The dropout-affected data were created using methods proposed in Huang et al. (15) (1st row), Tang et al. (16) (2nd row) and Chen et al. (17) (3rd row). The reference data was sampled at 0.5% efficiency for Huang method, divided by 500 for Tang method, and 25% of the nonzero data element was randomly filled with zero following Tang et al. The rates of creating zeros was chosen for these techniques in such a way that the osberved data contains roughly 70% zeros in all three methods. The reference data was build by taking the most informative 12,162 cells and 2,422 genes (see Methods). The reference data had 62% zeros, whereas the observed data had 68.4% (Huang), 68.7% (Tang), and 71.5% (Chen) zeros. for The spread of the violin denotes the standard deviation of the coefficient values across different cells.

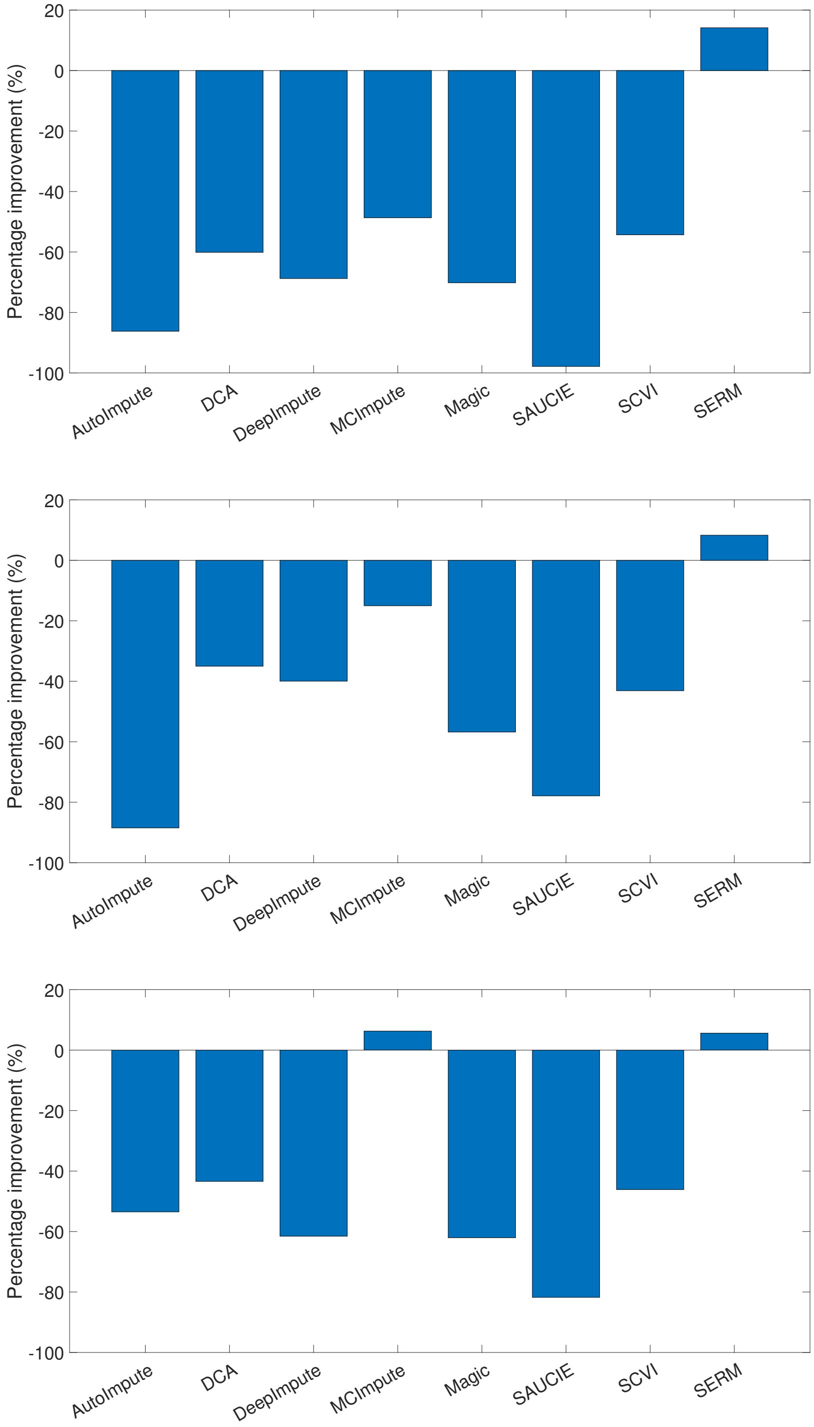

Fig. S52. Percent improvement of the Pearson coefficient in the imputed data by eight different techniques for cellular taxonomy dataset. The observed data were created (see Fig. S51) using methods proposed in Huang et al. (15) (1st row), Tang et al. (16) (2nd row) and Chen et al. (17) (3rd row).

Supplementary References

1. Andrew S. Venteicher, Itay Tirosh, Christine Hebert, Keren Yizhak, Cyril Neftel, Mariella G. Filbin, Volker Hovestadt, Leah E. Escalante, McKenzie L. Shaw, Christopher Rodman, Shawn M. Gillespie, Danielle Dionne, Christina C. Luo, Hiranmayi Ravichandran, Ravindra Mylvaganam, Christopher Mount, Maristela L. Onozato, Brian V. Nahed, Hiroaki Wakimoto, William T. Curry, A. John Iafrate, Miguel N. Rivera, Matthew P. Frosch, Todd R. Golub, Priscilla K. Brastianos, Gad Getz, Anoop P. Patel, Michelle Monje, Daniel P. Cahill, Orit Rozenblatt-Rosen, David N. Louis, Bradley E. Bernstein, Aviv Regev, and Mario L. Suvà. Decoupling genetics, lineages, and microenvironment in IDH-mutant gliomas by single-cell RNA-seq. 355(6332):eaai8478. . URL https://www.science.org/doi/10.1126/science.aai8478.
2. Mariella G. Filbin, Itay Tirosh, Volker Hovestadt, McKenzie L. Shaw, Leah E. Escalante, Nathan D. Mathewson, Cyril Neftel, Nelli Frank, Kristine Pelton, Christine M. Hebert, Christine Haberler, Keren Yizhak, Johannes Gojo, Kristof Egervari, Christopher Mount, Peter van Galen, Dennis M. Bonal, Quang-De Nguyen, Alexander Beck, Claire Sinai, Thomas Czech, Christian Dorfer, Liliana Goumnerova, Cinzia Lavarino, Angel M. Carcaboso, Jaume Mora, Ravindra Mylvaganam, Christina C. Luo, Andreas Peyrl, Mara Popović, Amedeo Azizi, Tracy T. Batchelor, Matthew P. Frosch, Maria Martinez-Lage, Mark W. Kieran, Pratiti Bandopadhayay, Rameen Beroukhim, Gerhard Fritsch, Gad Getz, Orit Rozenblatt-Rosen, Kai W. Wucherpfennig, David N. Louis, Michelle Monje, Irene Slavc, Keith L. Ligon, Todd R. Golub, Aviv Regev, Bradley E. Bernstein, and Mario L. Suvà. Developmental and oncogenic programs in H3K27M gliomas dissected by single-cell RNA-seq. 360 (6386):331–335. . URL https://www.science.org/doi/10.1126/science.aao4750.
3. Itay Tirosh, Benjamin Izar, Sanjay M. Prakadan, Marc H. Wadsworth, Daniel Treacy, John J. Trombetta, Asaf Rotem, Christopher Rodman, Christine Lian, George Murphy, Mohammad Fallahi-Sichani, Ken Dutton-Regester, Jia-Ren Lin, Ofir Cohen, Parin Shah, Diana Lu, Alex S. Genshaft, Travis K. Hughes, Carly G. K. Ziegler, Samuel W. Kazer, Aleth Gaillard, Kellie E. Kolb, Alexandra-Chloé Villani, Cory M. Johannessen, Aleksandr Y. Andreev, Eliezer M. Van Allen, Monica Bertagnolli, Peter K. Sorger, Ryan J. Sullivan, Keith T. Flaherty, Dennie T. Frederick, Judit Jané-Valbuena, Charles H. Yoon, Orit Rozenblatt-Rosen, Alex K. Shalek, Aviv Regev, and Levi A. Garraway. Dissecting the multicellular ecosystem of metastatic melanoma by single-cell RNA-seq. 352(6282):189–196. . URL https://www.science.org/doi/10.1126/science.aad0501.
4. Naomi Habib, Yinqing Li, Matthias Heidenreich, Lukasz Swiech, Inbal Avraham-Davidi, John J. Trombetta, Cynthia Hession, Feng Zhang, and Aviv Regev. Div-Seq: Single-nucleus RNA-Seq reveals dynamics of rare adult newborn neurons. 353(6302):925–928. ISSN 0036-8075, 1095-9203. . URL https://science.sciencemag.org/content/353/6302/925.
5. Heping Xu, Jiarui Ding, Caroline B. M. Porter, Antonia Wallrapp, Marcin Tabaka, Sai Ma, Shujie Fu, Xuanxuan Guo, Samantha J. Riesenfeld, Chienwen Su, Danielle Dionne, Lan T. Nguyen, Ariel Lefkovith, Orr Ashenberg, Patrick R. Burkett, Hai Ning Shi, Orit Rozenblatt-Rosen, Daniel B. Graham, Vijay K. Kuchroo, Aviv Regev, and Ramnik J. Xavier. Transcriptional Atlas of Intestinal Immune Cells Reveals that Neuropeptide -CGRP Modulates Group 2 Innate Lymphoid Cell Responses. 51(4):696–708.e9, . ISSN 1074-7613. .
6. Åsa Segerstolpe, Athanasia Palasantza, Pernilla Eliasson, Eva-Marie Andersson, Anne-Christine Andréasson, Xiaoyan Sun, Simone Picelli, Alan Sabirsh, Maryam Clausen, Magnus K. Bjursell, David M. Smith, Maria Kasper, Carina Ämmälä, and Rickard Sandberg. Single-Cell Transcriptome Profiling of Human Pancreatic Islets in Health and Type 2 Diabetes. 24(4):593–607. ISSN 1550-4131. . URL https://www.sciencedirect.com/science/article/pii/S1550413116304363.
7. Maayan Baron, Adrian Veres, Samuel L. Wolock, Aubrey L. Faust, Renaud Gaujoux, Amedeo Vetere, Jennifer Hyoje Ryu, Bridget K. Wagner, Shai S. Shen-Orr, Allon M. Klein, Douglas A. Melton, and Itai Yanai. A Single-Cell Transcriptomic Map of the Human and Mouse Pancreas Reveals Inter- and Intra-cell Population Structure. 3(4):346–360.e4. ISSN 2405-4712. . URL https://www.sciencedirect.com/science/article/pii/S2405471216302666.
8. Mauro J. Muraro, Gitanjali Dharmadhikari, Dominic Grün, Nathalie Groen, Tim Dielen, Erik Jansen, Leon van Gurp, Marten A. Engelse, Francoise Carlotti, Eelco J. P. de Koning, and Alexander van Oudenaarden. A Single-Cell Transcriptome Atlas of the Human Pancreas. 3(4):385–394.e3. ISSN 2405-4712. . URL https://www.sciencedirect.com/science/article/pii/S2405471216302927.
9. Yurong Xin, Jinrang Kim, Haruka Okamoto, Min Ni, Yi Wei, Christina Adler, Andrew J. Murphy, George D. Yancopoulos, Calvin Lin, and Jesper Gromada. RNA Sequencing of Single Human Islet Cells Reveals Type 2 Diabetes Genes. 24(4):608–615. ISSN 1550-4131. . URL https://www.sciencedirect.com/science/article/pii/S155041311630434X.
10. Yue J. Wang, Jonathan Schug, Kyoung-Jae Won, Chengyang Liu, Ali Naji, Dana Avrahami, Maria L. Golson, and Klaus H. Kaestner. Single-Cell Transcriptomics of the Human Endocrine Pancreas. 65(10):3028–3038. ISSN 0012-1797. . URL https://doi.org/10.2337/db16-0405.
11. Hoa Thi Nhu Tran, Kok Siong Ang, Marion Chevrier, Xiaomeng Zhang, Nicole Yee Shin Lee, Michelle Goh, and Jinmiao Chen. A benchmark of batch-effect correction methods for single-cell RNA sequencing data. 21(1):12. ISSN 1474-760X. . URL https://doi.org/10.1186/s13059-019-1850-9.
12. Xinyi Xu, Xiaokang Yu, Gang Hu, Kui Wang, Jingxiao Zhang, and Xiangjie Li. Propensity score matching enables batch-effect-corrected imputation in single-cell RNA-seq analysis. 23(4):bbac275, . ISSN 1477-4054. . URL https://doi.org/10.1093/bib/bbac275.
13. Ilya Korsunsky, Nghia Millard, Jean Fan, Kamil Slowikowski, Fan Zhang, Kevin Wei, Yuriy Baglaenko, Michael Brenner, Po-ru Loh, and Soumya Raychaudhuri. Fast, sensitive and accurate integration of single-cell data with Harmony. 16(12):1289–1296. ISSN 1548-7105. . URL https://www.nature.com/articles/s41592-019-0619-0.
14. Nicholas Schaum, Jim Karkanias, Norma F. Neff, Andrew P. May, Stephen R. Quake, Tony Wyss-Coray, Spyros Darmanis, Joshua Batson, Olga Botvinnik, Michelle B. Chen, Steven Chen, Foad Green, Robert C. Jones, Ashley Maynard, Lolita Penland, Angela Oliveira Pisco, Rene V. Sit, Geoffrey M. Stanley, James T. Webber, Fabio Zanini, Ankit S. Baghel, Isaac Bakerman, Ishita Bansal, Daniela Berdnik, Biter Bilen, Douglas Brownfield, Corey Cain, Michelle B. Chen, Steven Chen, Min Cho, Giana Cirolia, Stephanie D. Conley, Spyros Darmanis, Aaron Demers, Kubilay Demir, Antoine de Morree, Tessa Divita, Haley du Bois, Laughing Bear Torrez Dulgeroff, Hamid Ebadi, F. Hernán Espinoza, Matt Fish, Qiang Gan, Benson M. George, Astrid Gillich, Foad Green, Geraldine Genetiano, Xueying Gu, Gunsagar S. Gulati, Yan Hang, Shayan Hosseinzadeh, Albin Huang, Tal Iram, Taichi Isobe, Feather Ives, Robert C. Jones, Kevin S. Kao, Guruswamy Karnam, Aaron M. Kershner, Bernhard M. Kiss, William Kong, Maya E. Kumar, Jonathan Y. Lam, Davis P. Lee, Song E. Lee, Guang Li, Qingyun Li, Ling Liu, Annie Lo, Wan-Jin Lu, Anoop Manjunath, Andrew P. May, Kaia L. May, Oliver L. May, Ashley Maynard, Marina McKay, Ross J. Metzger, Marco Mignardi, Dullei Min, Ahmad N. Nabhan, Norma F. Neff, Katharine M. Ng, Joseph Noh, Rasika Patkar, Weng Chuan Peng, Lolita Penland, Robert Puccinelli, Eric J. Rulifson, Nicholas Schaum, Shaheen S. Sikandar, Rahul Sinha, Rene V. Sit, Krzysztof Szade, Weilun Tan, Cristina Tato, Krissie Tellez, Kyle J. Travaglini, Carolina Tropini, Lucas Waldburger, Linda J. van Weele, Michael N. Wosczyna, Jinyi Xiang, Soso Xue, Justin Youngyunpipatkul, Fabio Zanini, Macy E. Zardeneta, Fan Zhang, Lu Zhou, Ishita Bansal, Steven Chen, Min Cho, Giana Cirolia, Spyros Darmanis, Aaron Demers, Tessa Divita, Hamid Ebadi, Geraldine Genetiano, Foad Green, Shayan Hosseinzadeh, Feather Ives, Annie Lo, Andrew P. May, Ashley Maynard, Marina McKay, Norma F. Neff, Lolita Penland, Rene V. Sit, Weilun Tan, Lucas Waldburger, Justin Youngyunpipatkul, Joshua Batson, Olga Botvinnik, Paola Castro, Derek Croote, Spyros Darmanis, Joseph L. DeRisi, Jim Karkanias, Angela Oliveira Pisco, Geoffrey M. Stanley, James T. Webber, Fabio Zanini, Ankit S. Baghel, Isaac Bakerman, Joshua Batson, Biter Bilen, Olga Botvinnik, Douglas Brownfield, Michelle B. Chen, Spyros Darmanis, Kubilay Demir, Antoine de Morree, Hamid Ebadi, F. Hernán Espinoza, Matt Fish, Qiang Gan, Benson M. George, Astrid Gillich, Xueying Gu, Gunsagar S. Gulati, Yan Hang, Albin Huang, Tal Iram, Taichi Isobe, Guruswamy Karnam, Aaron M. Kershner, Bernhard M. Kiss, William Kong, Christin S. Kuo, Jonathan Y. Lam, Benoit Lehallier, Guang Li, Qingyun Li, Ling Liu, Wan-Jin Lu, Dullei Min, Ahmad N. Nabhan, Katharine M. Ng, Patricia K. Nguyen, Rasika Patkar, Weng Chuan Peng, Lolita Penland, Eric J. Rulifson, Nicholas Schaum, Shaheen S. Sikandar, Rahul Sinha, Krzysztof Szade, Serena Y. Tan, Krissie Tellez, Kyle J. Travaglini, Carolina Tropini, Linda J. van Weele, Bruce M. Wang, Michael N. Wosczyna, Jinyi Xiang, Hanadie Yousef, Lu Zhou, Joshua Batson, Olga Botvinnik, Steven Chen, Spyros Darmanis, Foad Green, Andrew P. May, Ashley Maynard, Angela Oliveira Pisco, Stephen R. Quake, Nicholas Schaum, Geoffrey M. Stanley, James T. Webber, Tony Wyss-Coray, Fabio Zanini, Philip A. Beachy, Charles K. F. Chan, Antoine de Morree, Benson M. George, Gunsagar S. Gulati, Yan Hang, Kerwyn Casey Huang, Tal Iram, Taichi Isobe, Aaron M. Kershner, Bernhard M. Kiss, William Kong, Guang Li, Qingyun Li, Ling Liu, Wan-Jin Lu, Ahmad N. Nabhan, Katharine M. Ng, Patricia K. Nguyen, Weng Chuan Peng, Eric J. Rulifson, Nicholas Schaum, Shaheen S. Sikandar, Rahul Sinha, Krzysztof Szade, Kyle J. Travaglini, Carolina Tropini, Bruce M. Wang, Kenneth Weinberg, Michael N. Wosczyna, Sean M. Wu, Hanadie Yousef, Ben A. Barres, Philip A. Beachy, Charles K. F. Chan, Michael F. Clarke, Spyros Darmanis, Kerwyn Casey Huang, Jim Karkanias, Seung K. Kim, Mark A. Krasnow, Maya E. Kumar, Christin S. Kuo, Andrew P. May, Ross J. Metzger, Norma F. Neff, Roel Nusse, Patricia K. Nguyen, Thomas A. Rando, Justin Sonnenburg, Bruce M. Wang, Kenneth Weinberg, Irving L. Weissman, Sean M. Wu, Stephen R. Quake, Tony Wyss-Coray, The Tabula Muris Consortium, Overall coordination, Logistical coordination, Organ collection and processing, Library preparation and sequencing, Computational data analysis, Cell type annotation, Writing group, Supplemental text writing group, and Principal investigators. Single-cell transcriptomics of 20 mouse organs creates a Tabula Muris. 562(7727):367–372. ISSN 1476-4687. . URL https://www.nature.com/articles/s41586-018-0590-4.
15. Mo Huang, Jingshu Wang, Eduardo Torre, Hannah Dueck, Sydney Shaffer, Roberto Bonasio, John I. Murray, Arjun Raj, Mingyao Li, and Nancy R. Zhang. SAVER: Gene expression recovery for single-cell RNA sequencing. 15(7):539–542. ISSN 1548-7105. .
16. Wenhao Tang, François Bertaux, Philipp Thomas, Claire Stefanelli, Malika Saint, Samuel Marguerat, and Vahid Shahrezaei. bayNorm: Bayesian gene expression recovery, imputation and normalization for single-cell RNA-sequencing data. 36(4):1174–1181. ISSN 1367-4803. . URL https://doi.org/10.1093/bioinformatics/btz726.
17. Mengjie Chen and Xiang Zhou. VIPER: Variability-preserving imputation for accurate gene expression recovery in single-cell RNA sequencing studies. 19(1):196. ISSN 1474-760X. .
